# Optimizing solar and wind forecasting with iHow optimization algorithm and multi-scale attention networks

**DOI:** 10.1038/s41598-026-39632-y

**Published:** 2026-03-10

**Authors:** Marwa Radwan, Abdelhameed Ibrahim, Mohamed M. Abdelsalam, Amel Ali Alhussan, Ebrahim A. Mattar, El-Sayed M. El-Kenawy

**Affiliations:** 1 Faculty of Artificial Intelligence, Delta University for Science and Technology, Mansoura, 11152 Egypt; 2 Computer Engineering and Control Systems Department, Faculty of Engineering, Mansoura University, Mansoura, 35516 Egypt; 3 Faculty of Engineering, Mansoura National University, Gamasa, Egypt; 4 Department of Communications and Electronics, Delta Higher Institute of Engineering and Technology, Mansoura, 35111 Egypt; 5 Department of Computer Sciences, College of Computer and Information Sciences, Princess Nourah bint Abdulrahman University, Riyadh, 11671 P.O. Box 84428, Saudi Arabia; 6 College of Engineering, University of Bahrain, Isa Town, Bahrain

**Keywords:** Solar and wind forecasting, Deep learning optimization, iHow optimization algorithm, Feature selection algorithms, Renewable energy prediction, Energy science and technology, Engineering, Mathematics and computing

## Abstract

Deep learning models often encounter two key challenges in developing intelligent and scalable forecasting frameworks for renewable energy systems: input feature space dimensionality and sensitivity to hyperparameter settings. These limitations increase computational cost and compromise generalization and robustness. This paper presents a hybrid deep learning–optimization framework that leverages cognitively inspired metaheuristics to address these challenges, employing the **Binary iHow Optimization Algorithm (biHOW)** for feature selection and its continuous counterpart, **iHOW**, for hyperparameter tuning. Both variants emulate human cognitive phases—data absorption, information analysis, reinstitution, and adaptive knowledge development enabling efficient traversal of complex search spaces. Using the **Multi-Scale Attention Network (MSAN)** as the forecasting backbone, which is well suited for modeling renewable energy time series due to its ability to capture multi-scale temporal dependencies ranging from short-term fluctuations to long-term seasonal patterns, the proposed framework achieved high accuracy for wind and solar generation prediction. The MSAN model attained Mean Squared Errors (MSE) of 0.0105 for wind and 0.0976 for solar forecasting. Applying biHOW for feature selection reduced the average misclassification rate to 0.3925 (wind) and 0.4161 (solar) while identifying compact, interpretable feature subsets. The iHOW optimizer further fine-tuned architectural and training parameters, decreasing MSE to $1.10883\times 10^{-6}$ for wind and $7.08664\times 10^{-6}$ for solar, outperforming state-of-the-art metaheuristics including HHO, GWO, PSO, and JAYA. These findings demonstrate the effectiveness of iHOW-based optimization in enhancing forecasting accuracy and computational scalability. The proposed hybrid framework supports adaptive forecasting for intelligent energy management within modern smart grids.

## Introduction

Due to the growing global demand to shift toward sustainable energy systems, renewable energy resources are critical foundations in the quest against climate change, energy security domination, and long-term ecological balance^1–3^. Solar and wind, two of the wide range of renewables, have received unprecedented interest because they scale well, are abundant and have a very low levelized cost of electricity (LCOE) because of the maturing of solar and wind technology^4–6^. Their use is very effective in curbing the production of greenhouse gases and alleviating the effects that the environment would have suffered as a result of burning fossil fuels^7,8^. Although solar and wind technologies offer numerous benefits to consumers, their variable and intermittent nature makes it challenging to integrate them into modern power systems^9,10^. Conditions of energy production are variable because they depend on weather conditions (clouds, irradiance, wind turbulence), creating uncertainties in forecasting energy production, scheduling energy production generation, and maintenance schedules. To circumvent these uncertainties, there has been a growing need in recent years to implement predictive maintenance approaches in research and even industry. By definition, predictive maintenance is concerned with predicting the probability of equipment failure or deterioration as it is bound to happen. The proactive measure prevents unscheduled outages, minimizes expenditure on maintenance, maximizes machine life and increases overall work efficiency. Within the context of solar and wind energy systems, predictive maintenance is particularly important because distributed components (e.g., photovoltaic [PV] panels, inverters, wind turbines and power converters) are often deployed in large numbers in a highly variable mechanical and electrical environment^11^. One of the most critical drivers of predictive maintenance in the last few years has been the development of machine learning (ML)^12,13^ and deep learning (DL) methods. With these methods, complicated and non-linear relations can be modeled using vast amounts of past observations of the sensor. Unlike traditional analytical models, which rely on clear physical equations or hand-designed jobs, DL models can autonomously discover latent patterns in raw time series. Examples of architecture include recurrent neural networks (RNNs), long short-term memory networks (LSTM), and attention-based mechanisms, which have proven highly effective in the time series forecasting task. They can be applied to predict failure risks and performance trends in renewable energy systems because long-term dependencies, multi-scale temporal patterns, and dynamic contextual shifts can be learned with their help^14^. Recent studies further highlight that renewable energy forecasting and broader energy-management prediction tasks increasingly require (i) principled feature evaluation/selection to mitigate redundancy in high-dimensional settings and (ii) architectures capable of capturing complex temporal dependencies. For example, Huang et al.^15^ proposed a hybrid multivariate time-series prediction system that combines transfer entropy with density-aware learning to uncover relationships among variables and improve modeling efficiency. In addition, Cai et al.^16^ developed an interval prediction framework for energy management that integrates dynamic feature selection with frequency-domain learning and sparse-attention-enhanced LSTM modeling, emphasizing both redundancy control and temporal expressiveness. Related efforts also stress the difficulty of adapting deep architectures: Wu and Zhan^17^ proposed an adaptive CNN–Transformer fusion model with discriminative fuzzy clustering to determine architectural components more effectively, while a recent survey by Zhan et al.^18^ summarizes how fuzzy information granules can reduce dimensionality and enhance interpretability in neural prediction. Beyond forecasting, optimization-based decision support has also been studied in large-scale settings; for instance, Zhan and Cai^19^ introduced a cost-minimized consensus mechanism under dynamic trust networks, illustrating the broader importance of optimization under uncertainty. These studies collectively motivate the need for an integrated forecasting framework that jointly improves feature representation and model configuration under complex temporal dynamics, which is the focus of the proposed MSAN–(biHOW/iHOW) pipeline.

Despite the prospect of predictive models powered by DL, multiple issues complicate the successful incorporation of DL in solar and wind forecasting. Among the most prominent concerns, high dimensionality emerges as a challenging problem to cope with that could be brought about by the high utilization of sensor networks in the field of contemporary renewable energy infrastructure. They can log many different features such as temperature, pressure, irradiance, humidity, vibration, voltage, and current, among others. Although this amount of data is rich in representing the possibility of fine-tuned knowledge, it tends to introduce redundancies and other irrelevant information that might interfere with the model’s performance^20^. The high-dimensional feature space is not only a demanding aspect of computing, but it further compounds the risk of overfitting, particularly when there is limited data to label. Further, collinearity or irrelevance of variance in many of the sensor signals can hide functional patterns. Hence, efficient attribute selection turns out to be a mandatory requirement to develop reliable and understandable forecasting models. Furthermore, the incompleteness of data, class imbalance with failure labels, and non-stationary distributions also increase the complexity of model development. This paper aims to research and review deep learning techniques in a mannerized way within deep learning models, enabling the accurate and scalable prediction of solar and wind energy forecasting. The main task has a two-fold nature, namely, on the one hand, to evaluate a range of state-of-the-art DL structures with the ability to extract the temporal dynamics involved in the renewable energy production; on the other hand, to utilize metaheuristic optimization methods to improve these models. Models discussed include Multi-Scale Attention Network (MSAN), Long Short-Term Memory network (LSTM), Gated Recurrent Unit (GRU), Generative Adversarial Network for Time Series (GANT), and Adaptive Residual Network (ARN), chosen for their relevance to time series modeling and forecasting tasks. In this study, the design of the MSAN model is of particular importance because it has been identified as the general forecasting architecture. An alternative metaheuristic algorithm, the iHow Optimization Algorithm (iHow) is suggested to solve two key problems of renewable energy forecasting: feature selection and hyperparameter optimization. This optimizer can be used to optimize the training of the Multi-Scale Attention Network (MSAN) by optimizing its architectural parameters, e.g. number of layers, attention heads and dropout rates, and the feature set it is fed on. The overall goal of the proposed paper is to obtain the best possible predictive accuracy, which will be measured using traditional regression indicators such as the Mean Squared Error (MSE), Root Mean Squared Error (RMSE), and Coefficient of Determination ($R^2$). The proposed research design introduces a new forecasting pipeline for renewable energy generation that integrates deep learning with metaheuristic optimization. Rather than proposing a new optimizer, this work focuses on the effective *utilization* of an existing cognitively inspired optimization algorithm within a carefully designed hybrid forecasting framework for wind and solar energy prediction. Specifically, the framework combines deep learning models with metaheuristic-driven feature selection and hyperparameter tuning to improve predictive accuracy, robustness, and computational efficiency in renewable energy forecasting. The main contributions of this work are summarized as follows:

- We adopt the cognitively inspired **iHow Optimization Algorithm (iHow)**, originally proposed in prior work, and integrate it into a renewable energy forecasting framework. The algorithm is conceptually inspired by human cognitive learning processes such as memory formation, information processing, and behavioral adaptation. Its role in this study is to efficiently guide the optimization process by balancing global exploration and local exploitation in complex, high-dimensional search spaces.
- Our algorithm can be fractionalised into a binary form, **Binary (biHOW)**, implementing a proper feature selection. In this work, the binary variant is employed to select informative input features, enabling dimensionality reduction without sacrificing forecasting accuracy.
- We combine the iHOW framework and the **Multi-Scale Attention Network (MSAN)** for automatic hyperparameter tuning, demonstrating how an existing cognitive-based optimizer can be effectively coupled with a multi-scale attention architecture to improve convergence stability, robustness, and predictive performance.
- We create a multi-dimensional forecasting system specifically optimised for wind and solar energy generation based on actual French grid datasets. The proposed framework leverages temporal and categorical features to capture the inherent non-stationary and seasonal characteristics of renewable energy production.
- We conduct a thorough empirical analysis, scoring our proposed framework alongside several state-of-the-art meta-heuristic algorithms, namely Harris Hawks Optimization (HHO), Grey Wolf Optimizer (GWO), Particle Swarm Optimization (PSO), Whale Optimization Algorithm (WAO), Biogeography-Based Optimizer (BBO), Multi-Verse Optimizer (MVO), Stochastic Fractal Search (SFS), Simulated Annealing Optimizer (SAO) and JAYA, to name a few, in order to assess the effectiveness of the proposed integration strategy rather than the invention of a new optimization mechanism.
- We demonstrate that deep learning models optimized using iHOW exhibit a consistent pattern of improved forecasting accuracy and computational efficiency. These results confirm that the proposed hybrid framework enables efficient and reliable renewable energy forecasting when an advanced optimizer is appropriately embedded within the learning pipeline.

The remainder of this paper is organized as follows. Section 2 reviews the existing literature, highlighting recent trends in metaheuristic optimization—particularly in human-inspired algorithms—and identifies the limitations of current procedures in both feature selection and predictive modeling for renewable energy systems, with a focus on solar and wind power. Section 3 details the materials and methods employed, including the dataset, preprocessing steps, deep learning architecture, and the proposed optimization framework. It also describes the role of the **Binary iHow Optimization Algorithm (biHow)** in feature selection and the use of the **iHow Optimization Algorithm (iHow)** for hyperparameter tuning. Section 4 presents the experimental results and a comparative analysis of the performance of the MSAN model and other deep learning architectures, both before and after optimization, benchmarking iHow against other state-of-the-art metaheuristics. Section 5 discusses the observed performance trends, practical implications, and insights into how the effective use of iHow within the proposed framework enhances forecasting accuracy and robustness. Finally, Sect. 6 concludes the paper and outlines prospective research directions, including the extension of the framework to real-time forecasting systems, ensemble prediction strategies, and adaptive energy management applications.

## Literature review

It is necessary that correct predictions of renewable energy resources particularly solar and wind energy will also play a significant role in overcoming the problems of variability and fluctuations of these resources. Literature has recently provided various methods and models to enhance the performance of prediction and make them easily applicable to smart grids. The prediction of the solar and wind resources in the Doomadgee region of Far North Queensland that has been faced with difficulty in predictions is likely to employ the Prophet-model-based method. The Prophet model had fewer errors, with a root mean squared error (RMSE) of 0.284 and a mean absolute error (MAE) of 0.394 for solar data, and an RMSE of 0.527. The MAE was 0.427 in the case of the wind, illustrating better fitting prediction properties compared to the SARIMA model^21^. Further accuracy in prediction will be achieved by developing an Artificial Neural Network (ANN) optimization optimized using the Harmony Search Algorithm (HSA). In this model, ANN connections have optimized weights that give high performance in forecasting both solar and wind energies. As an example, HSA-optimized ANN was able to land on RMSE of 0.21805 and MAE of 0.18546 regarding solar irradiation and RMSE of 0.55627 and MAE of 0.47172 in wind speed, making it better than traditional ANN methods^22^. Introducing Data Assimilation (DA) into solar wind time series forecasting results in an improved near-Earth prediction, as refined inner boundary conditions based on spacecraft data, such as STEREO and ACE, are combined with a variational Data Assimilation scheme and the HUXt solar wind model. This strategy resulted in a reduction in the RMSE of 27-day predictions and increased the precision of prediction, particularly when predictions were made using corotation techniques that were constrained by latitudinal offsets^23^. A powerful type, ASTGNN-LSTM, which combines the convergence of attention-based spatial-temporal graph neural networks and LSTM, has been found effective in coping with the nonlinearities of 20 years of meteorological data belonging to Northwest China. It performed exceptionally well compared to standard models like ARIMA and basic LSTM set-up, with relative errors of 27.15% in wind speed and 6.11% in solar radiation^24^. Deep learning has enhanced the stability of microgrid (MG) through its incorporation in multi-step forecasting. A model predicting solar and wind power on MG systems using an LSTM-based system was developed, thereby resolving the vanishing gradient problem of shallow neural networks. This enabled load frequency regulation and improved coordination of nonrenewable resources through PI controllers^25^. The next generation of deep learning involves hybrid networks, such as HCLNet (CNN-LSTM) and HAELNet (Autoencoder-LSTM), which enhance solar energy forecasting in smart grids. HAELNet produced the best MAPEs for some of the most critical data, such as daily power and solar radiance, which underlines its higher ability to find x y long-term dependencies and intricate trends^26^. The second-order derivative grey prediction model has been used to achieve advancement in the prediction of wind energy in China, optimized with Particle Swarm Optimization. The model approximated wind-generated power to be 2425.44 billion kWh by 2030, providing helpful information for policy and planning. This was achieved through the reduction of data noise using a grey buffer operator and improvements in prediction by use of three-parameter whitening^27^. To overcome the issue of real-time prediction, a solution is proposed in the form of a dual-stage Kalman filtering with deep residual learning (DRL-Bi-LSTM). It decouples trend and residual components, separately modeling them, which results in MAE and RMSE values of 0.48 and 0.59, respectively. The model outperforms ARIMA, SVM, and LSTM, and it is adaptive to changing scenarios in real time^28^. An example of this is the STE-HOLNet, a new and radical deep learning technique that incorporates feature enhancement for both spatio-temporal phenomena and concept drifts into the system. Inventions such as E-Time2Vec encoding, TimesNetV2, and Inception modules, along with adaptive Mamba-based learning, slashed RMSE by 36.93%, MAE by 41.26%, and SMAPE by 56.57%, and were adaptive and accurate^29^. It is proposed that a combination of forecasting models, incorporating data denoising, ensemble techniques, and classical models, could enhance prediction accuracy. Applying to measure on 10-minute interval wind speed readings of Penglai, China, this method outperforms individual prediction methods because a multiplicity of perspectives are incorporated into a unified model^30^. With the help of two-stage feature selection and a Temporal Fusion Transformer (TFT), which is optimized by a Snow Ablation Optimizer (SAO), another promising route is the appearance of a hybrid model. The given method guarantees a high quality of the input features and multidimensional interpretability with tree SHAP, reaching the level of the MAPEs of 6.29 % on the Williams Wind Farm dataset season-to-season^31^. To advance the transformer-based method, the hybrid model, which combines physics-informed modeling and TFT, has exhibited a considerable increase in the accuracy of wind power forecasting. The model utilizes the twin Numerical Weather Prediction (NWP) observation records and ideal power curves to develop a Forecast Skill Index (FSI-WPF) against which the comparison may be conducted. It attains a maximum of up to 60 percent RMSE improvement and precision in the forecasting^32^. Wind and solar data were used to evaluate the feasibility of green hydrogen production in Southeast Asia at the regional level. The study concludes that wind-driven electrolysis is more economical than solar in certain countries, such as the Philippines, supporting the importance of wind forecasting for hydrogen viability^33^. It is also essential to enhance data quality. We have supplied a multitask learning (MTL) framework utilized in short-term wind power forecasting, comprising a competent anomaly discovery section which is based on isolation forests as well as DBSCAN. MTL models apply the cleaned data to predict over multiple time horizons with high anomaly detection precision (90%) and R2 (0.95) of the predictions^34^. A hybrid model of Sparse Variational Gaussian Process (SVGP) and Noisy Input Gaussian Process (NIGP) has been presented to enhance the resilience of weather transitions of a model. This approach decomposes time-series data on wind speed into signal and noise, which all but eliminates the uncertainty in the factors that define input and significantly enhances precision^35^. There are special problems that cold waves pose to wind power prediction. A segmented forecasting approach has been suggested, where Graph Convolutional Networks (GCN) and BiGRU are applied to learn the fluctuation patterns during cold waves. Such conditions can be overcome with the hybrid LightGBM-Transformer method that increases forecasting accuracy^36^. A hybrid model of seasonal wind power has been developed using an SVMD-SLSTM. This model provides an MAPE of up to 1.255% in seasonal forecasts due to successive variational mode decomposition to extract features and stacked LSTM layers to learn a sequence of time^37^. Lastly, a CNN-BiLSTM-STA constructs protocols of the spatiotemporal focus that generate endurance against unsettled and incomplete statistics. The model yields better outcomes on datasets of Arizona and Texas as well, due to its use of correntropy-based loss and partial reinforcement optimization, thus showcasing its scalability and dependability^38^. Recent advances in feature selection have increasingly emphasized multi-objective formulations to balance predictive accuracy and feature subset compactness. Fu et al. ^39^ proposed MOFS-REPLS, a large-scale multi-objective feature selection algorithm based on real-valued encoding and a preference leadership strategy. Their method enhances population diversity and preserves high-quality non-dominated solutions, demonstrating strong performance on high-dimensional datasets.Similarly, Li et al. ^40^ introduced a multi-objective binary grey wolf optimization approach with a guided mutation strategy to improve exploration and avoid premature convergence, achieving an effective trade-off between classification accuracy and feature reduction.Beyond feature selection, multi-objective optimization has also been applied to model selection in online learning systems. Jin et al. ^41^ proposed a multi-objective model selection algorithm for online sequential ultimate learning machines, improving robustness and accuracy in dynamic environments.While these approaches explicitly optimize competing objectives, the present work adopts a computationally efficient single-objective optimization strategy focused on forecasting accuracy and stability in renewable energy systems, leaving multi-objective extensions as future work. In Table 1, a summary of recent studies on solar and wind energy forecasting, utilizing various artificial intelligence and statistical methods, is presented. All sources are classified according to the goal, methodology, and essential conclusions to signal the scope of directions and discoveries in the field. Such comparative analysis makes it easier to realize the state-of-the-art in renewable energy forecasting and appreciate the methodological trends and the level of modeling performances of various modeling strategies and applications.

**Table 1:** Summary of Key Studies on Renewable Energy Forecasting Methods and Outcomes.

Refs.	Objective	Methodology	Key Findings
slam et al.^21^	Forecast solar and wind in FNQ, Australia	Prophet and SARIMA time-series models	Prophet model achieved lower RMSE and MAE, showing better performance than SARIMA
Mohsin et al.^22^	Enhance ANN-based forecasting	Harmony Search Algorithm optimized ANN (HSA-ANN)	Achieved improved MSE and RMSE compared to conventional ANN
Lang et al.^23^	Improve solar wind forecasts using spacecraft data	Variational Data Assimilation (DA) with HUXt model and STEREO/ACE data	Reduced RMSE and improved boundary accuracy even off Earth’s orbital plane
Li et al.^24^	Predict wind speed and solar radiation	ASTGNN-LSTM using 20 years of meteorological data	Lower relative errors; location had highest prediction influence
Kumar et al.^25^	Improve stability of microgrids	Multi-step LSTM forecasting for wind and solar	Forecasting supports load-frequency control with reduced instability
Zafar et al.^26^	Improve solar forecasting accuracy	Hybrid HAELNet and HCLNet (Autoencoder + LSTM, CNN-LSTM)	HAELNet had lowest MAPE, capturing long-term dependencies
Mao et al.^27^	Predict long-term wind power in China	Optimized second-order grey prediction model	Forecast: 2.6$\times$ increase by 2030; aided by noise-reduction preprocessing
Ahmadi et al.^28^	Improve short-term wind forecasting	Kalman filter + DRL-BiLSTM + RNN fusion	Outperformed ARIMA, SVM, and Transformer hybrids; MAE = 0.48
Zhao et al.^29^	Real-time adaptive wind forecasting	STE-HOLNet with E-Time2Vec and PS-HBPMamba	Reduced RMSE by 36.93%, improved R2 and long-term performance
Ghaffar et al.^30^	Combine classic methods for wind forecasting	Denoising, ensemble of classical models, optimization algorithm	Outperformed individual forecasting techniques using real wind farm data
Wu et al.^31^	Forecast wind speed with interpretability	Two-stage feature selection + SAO + TFT + SHAP	Achieved seasonal MAPE between 6.29%–16.22%, with explainable features
Michalakopoulos et al.^32^	Combine physics with deep learning for wind power	TFT + theoretical power curves + FSI-WPF	60% RMSE reduction, 99.47% forecasting score over conventional models
Chowdhury et al.^33^	Evaluate green hydrogen feasibility in SE Asia	Energy-climate-cost model using solar and wind data	Philippines most viable; wind electrolysis more cost-effective than solar
Ma et al.^34^	Clean data and forecast short-term wind power	Anomaly detection + multitask learning + BiLSTM	90% anomaly detection; R2 = 0.95; strong multistep accuracy
Zhang et al.^35^	Model uncertainty during transitional weather	SVGP + Noisy Input Gaussian Process (NIGP)	Forecasting errors corrected; improved confidence in variable weather
Lin et al.^36^	Forecast during extreme cold waves	SeqVAE + GCN + BiGRU + LightGBM-Transformer	Accurate cold wave losses prediction using hybrid segmented learning
Shringi et al.^37^	Seasonal wind power forecasting	SVMD decomposition + stacked LSTM	Seasonal MAPE as low as 1.255%; effective at multi-scale pattern recognition
Abid et al.^38^	Robust spatial-temporal wind/solar prediction	CNN-BiLSTM with spatiotemporal attention and correntropy loss	Outperformed 3 benchmarks across Arizona and Texas datasets
Fu et al.^39^	Large-scale multi-objective feature selection.	MOFS-REPLS with real-valued encoding and preference leadership.	Selects compact feature subsets with high accuracy; outperforms state-of-the-art MOFS methods.
Li et al.^40^	Multi-objective binary feature selection improvement.	Binary GWO with guided mutation and diversity control.	Achieves better trade-offs between accuracy and feature reduction than competing optimizers.
Jin et al.^41^	Multi-objective model selection for online learning.	Feedback-compensated multi-objective model selection strategy.	Improves robustness and accuracy in online sequential learning systems.

### Research gaps and motivation

Despite notable progress in solar and wind forecasting, several gaps remain that limit the effectiveness of existing approaches in practical renewable energy systems. Many studies emphasize forecasting architectures such as recurrent, convolutional, and hybrid deep learning models, but provide limited mechanisms for managing high-dimensional input spaces, often resulting in increased computational cost, feature redundancy, and reduced interpretability. Moreover, although metaheuristic optimization techniques have been employed to enhance prediction performance, most existing work focuses on conventional forecasting models rather than explicitly optimizing multi-scale attention networks. The performance of such networks depends on interrelated architectural and training parameters, including temporal scale design, attention heads, embedding dimensions, and regularization, which are not efficiently handled by generic optimization strategies. In addition, commonly used optimizers such as PSO, GWO, HHO, and WOA may suffer from premature convergence or sensitivity to parameter settings when applied to non-convex and mixed optimization spaces that combine discrete feature selection with continuous hyperparameter tuning, a scenario typical in renewable energy forecasting. Finally, many studies treat feature selection and hyperparameter tuning as independent stages, overlooking their coupled influence on forecasting accuracy and robustness. Motivated by these limitations, this work proposes an integrated iHow–MSAN framework that combines biHOW for compact feature selection with iHOW for adaptive hyperparameter tuning, specifically designed to optimize multi-scale attention networks for stable and accurate solar and wind energy forecasting.

## Materials and methods

**Fig. 1:**
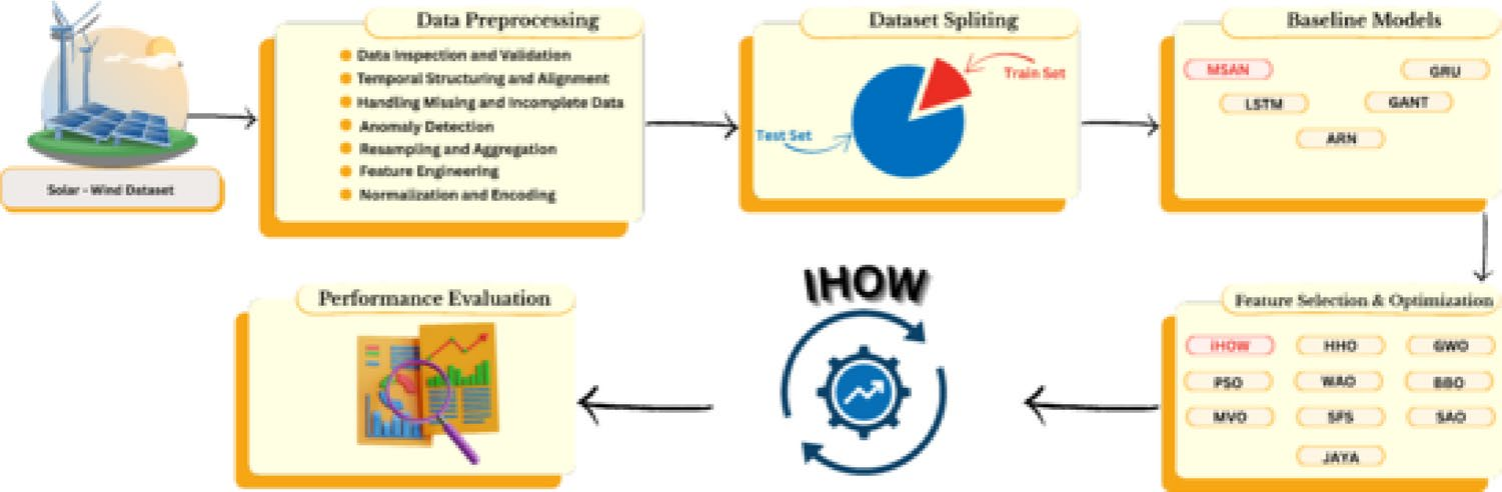
Proposed hybrid forecasting framework for solar and wind energy, integrating preprocessing, optimization, baseline modeling, and performance evaluation.

This paper presents a comprehensive approach that integrates data preprocessing, optimization strategy, baseline model evaluation, and performance evaluation, providing a systematic method for addressing the challenge of precise forecasting of renewable energy. The process, as presented in Fig. 1 presents the proposed hybrid forecasting framework designed to enhance the precision and robustness of solar–wind energy prediction. This is done through first collecting an integrated solar wind dataset; this is a set of meteorological and energy generated variables of renewable energy sources. During the data preprocessing stage, the raw data are systematically cleaned and structured, involving the inspection and verification of the data, temporal alignment, dealing with missing and incomplete values and detection of anomalies. Other processing activities like resampling, feature engineering, normalization, and encoding are used to guarantee data consistency and enhance generalization of the model. After preprocessing, the data will be split into training and testing groups to guarantee the absence of bias when testing models and avoid the issue of overfitting. A group of baseline deep learning models, which are Multi-Scale Attention Network (MSAN), Long Short-Term Memory (LSTM), Gated Recurrent Unit (GRU), Generative Adversarial Network of Time Series (GANT), and Attention Recurrent Network (ARN), are utilized to learn the temporal relationships and non-linear characteristics of the hybrid renewable energy data. Additional optimization algorithms based on metaheuristics are combined to improve the precision of the predictions, perform the feature selection, and optimize the hyperparameters. These will be the suggested Improved Harris Hawks Optimization (iHOW) algorithm, as well as competitive methods as HHO, GWO, PSO, MVO, SFS, BBO, WAO, SAO, and JAYA. Each optimizer sequentially explores the solution space to find the best model parameters and other useful features that will maximize predictive performance but reduce computational complexity. Lastly, the performance evaluation module measures the forecasting results quantitatively by evaluating several statistical performance measures, including: Root Mean Square Error (RMSE), Mean Absolute Error (MAE), Coefficient of Determination (R2). This analysis will make it possible to do a comparative analysis between the original models and their optimized counterparts, and therefore, establish the most efficient and precise forecasting model on integrated solar-wind system. On the whole, this hybrid framework offers a dynamic and systematic approach to data-driven modeling, which integrates with optimization-based intelligence in order to improve forecasting on renewable energy.

### Dataset description

The paper will use high-resolution data of hourly renewable generated energy (wind and solar) obtained from the French national grid. The data, spanning from 2020 to the present, supports regulatory efforts aimed at establishing a reference price by the French Law on Energy Transition for Green Growth (LTECV). It contains energy generation in megawatt-hours (MWh) with dense time-based metadata as well as source-type labeling. To maintain consistency of the experiments, the pre-prepared dataset is already split into two sets: Train.csv and Test.csv to train and validate the model, and to test how well the model generalizes. The dataset comprises records with specific production and timestamp attributes, including detailed production and timestamp characteristics, which are extracted to produce multiple-scale temporal patterns and modality-specific behaviors for intelligent forecasting. The main fields involved in this work are summed up in Table 2. These contain timestamp data, categorical variables related to the day and month, and the label of the energy source, all of which aid in the solar- and wind-specific model development. The composition, along with the variety of the dataset, makes it highly effective in terms of its ability to be modeled empirically, particularly through the use of elaborate deep learning models and metaheuristic optimization processes.

**Table 2:** Description of dataset features used for solar and wind energy forecasting.

Feature Name	Description
Date and Hour	Unified timestamp representing the date and hour of observation.
Date	Calendar date at daily granularity.
StartHour	Beginning of the measurement interval.
EndHour	End of the measurement interval.
Source	Energy source: wind or solar.
Production	Energy produced (in MWh), used as the forecasting target.
DayOfYear	Ordinal position of the day in the calendar year.
DayName	Name of the day (e.g., Monday).
MonthName	Month of the observation (e.g., January).

**Fig. 2:**
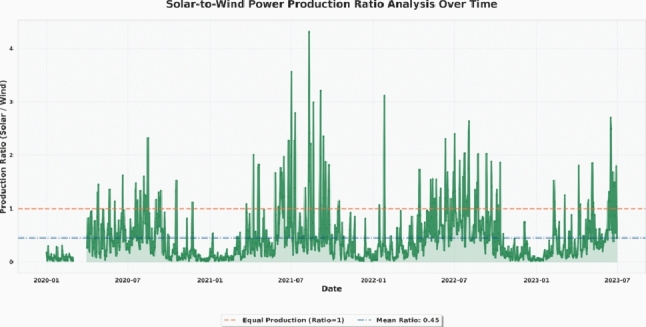
Temporal evolution of the solar-to-wind power production ratio. The orange dashed line represents the equal production threshold (Ratio = 1), and the blue dashed line indicates the mean ratio across the observed period.

The complementarity and balance of renewable energy resources cannot be evaluated without understanding the proportional relationship between the solar and wind power generation. Figure 2 illustrates the temporal evolution of the solar-to-wind production ratio throughout the observation period, providing insights into how the contributions of the two energy sources vary over time. The ratio is a key signifier of the dynamics of the energy mix by identifying the time when a single source dominates and also assesses the diversity of the entire system. As observed in Fig. 2, the mean production ratio across the dataset is approximately 0.45, indicating that, on average, solar generation constitutes less than half of the wind energy output. The orange dotted line at the ratio of 1.0 represents the point of equal production where the solar energy exceeds the wind output. Time series shows definite seasonal variations with the sun primarily dominating in summer seasons whereas the wind power is mostly dominant in cold seasons. These alternating dominance patterns highlight the complementary nature of the sun irradiance and wind velocity cycle, highlighting the fact that integrated hybrid forecasting and joint energy management systems may prove advantageous.

### Data preprocessing

The preprocessing process is a very important phase in the process of constructing strong and viable forecasting models in generation of renewable energy. The raw data obtained in real-life energy systems is usually characterized by discrepancies including missing values, irregular time stamps and non-uniform scales of features. Thus, a well-thought preprocessing pipeline was used to guarantee the quality of the data, its consistency, and availability before the deep learning models were trained.

#### Data inspection and validation

The data consisted of hourly wind and solar power plants in France. A preliminary validation was done to check the integrity of data, accuracy of the type of data and completeness of some of the key attributes like Date and Hour, Date, Source, and production. The absence or blank values in the field of Production were detected and examined to determine their commonality and trend. This validation exercise was made to guarantee that further transformations and feature engineering operations would be executed on a stable and error-free basis.

#### Temporal structuring and alignment

The date and hour field had to be transformed into a uniform time-series, which serves better than time-series analysis with dates; this was done by converting it into a time-aware, standardized, and standardized version of time-series (datetime utc=True). This conversion made it possible to perform temporal operations with high precision including resampling, lag generation and rolling statistics. The data were then restructured into a pivoted transformation with two parallel time series; one of solar energy production and the other of wind energy production whereby, the time index was created using the date and hour. Such format made possible parallel modelling and face-to-face comparison of the generation profiles of the two sources of energy. Lastly, the records were ordered in time order to maintain the continuity of time and eliminate the possibilities of misalignment in training the model.

#### Handling missing and incomplete data

Since the energy systems were operational, it was anticipated that data gaps were going to occur as a result of sensor malfunctioning or communication interruptions. These issues were dealt with in a two step method. Records that had completely missing values are discarded to avoid noise concentration and partially missing values are replaced through localized linear interpolation that was limited within the same day. In this way the intra-day transition was smooth and did not distort inter-day trends. These corrections maintained the coherence of time and statistical consistency needed to carry out the downstream learning activities.

**Fig. 3:**
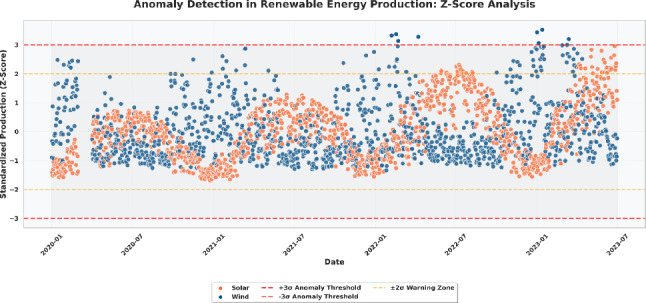
Anomaly detection in solar and wind energy production using Z-score analysis. The red dashed lines represent $\pm 3\sigma$ anomaly thresholds, while the yellow dashed lines indicate the $\pm 2\sigma$ warning zone.

To detect anomalies and extreme deviation on the generation of renewable energies, an anomaly detection mechanism which relies on Z-score was used on the standardized data of production. The analysis, presented in Fig. 3, highlights the deviations of solar and wind energy production from their respective mean values over the observation period. When the data on production is converted into uniform scores, then one can identify the abnormal occurrences, like sharp increase or decrease in production, which deviates out of the statistical expectations.

As shown in Fig. 3, most production values for both energy sources lie within the $\pm 2\sigma$ warning zone, indicating normal operational behavior. Some of these points however surpass the levels of the anomalies of the levels of the three sigma, which are the red worried lines, indicating that there are abnormal activity levels in production. These extremes are usually associated with sudden weather variations, faulty sensors or unusual working states. The figure also illustrates that wind energy has a more widespread distribution of anomalies than solar energy, which shows that it is more variable and relies more on atmospheric processes. The process of anomaly detection is necessary in both the maintenance of data quality and the enhancement of the strength of further predictive modeling in the renewable energy forecasting.

#### Resampling and aggregation

In order to examine larger-scale trends of time, the hourly data were resampled into daily data with a time-based summation (resample(’D’).sum()). The conversion minimized the high frequency noise and accentuated the long-term trends and seasonal variations in the production of solar and wind power. The resampled data presented a consistent foundation of analyzing the diurnal and seasonal reliances on renewable energy production.

#### Feature engineering

**Fig. 4:**
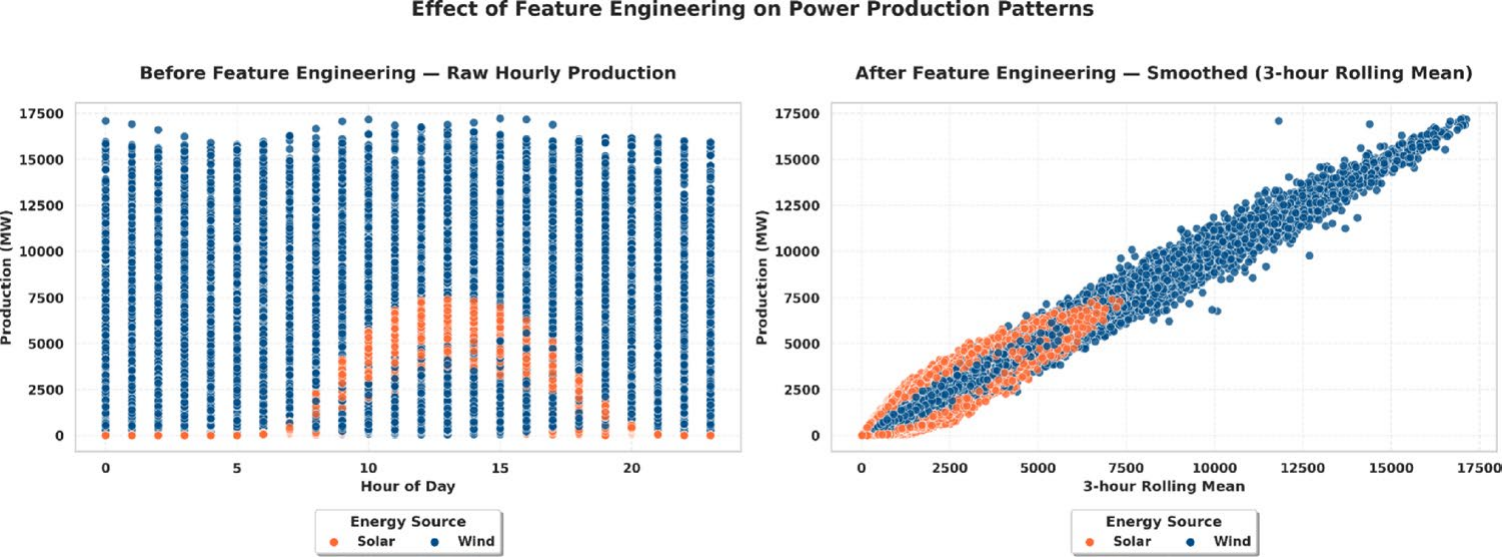
Effect of feature engineering on power production patterns. The left panel shows raw hourly production data for solar and wind energy, while the right panel presents smoothed data obtained through a three-hour rolling mean transformation.

The feature engineering was used to add some details to the dataset and boost the predictive abilities of the model. Some temporal and statistical characteristics were created, such as Lag-based characteristics like the past one-hour production ( Lag 1h ) to represent short-term characteristics and rolling characteristics like a three-hour moving average ( Rolling 3h ). Other derived values were the Solar to Wind Ratio, the relative contribution of each energy source and calendar based values like the Month Num, Weekday and Weekend values which represented seasonal and cyclical patterns in energy production. A fundamental measure in pre-treating the dataset to the deep learning-based forecasting was to investigate the impact of feature engineering on the time dynamics of solar and wind energy generation. As illustrated in Fig. 4, the transformation from raw hourly measurements to engineered features significantly altered the underlying structure of the data, improving its smoothness and correlation across time. Before feature engineering, the raw hourly production values displayed pronounced volatility and high-frequency noise, as shown in the left panel of Fig. 4. Such intermittent tendency was more pronounced in wind power data which had often short-term variations whereas solar production had high diurnal variations. This kind of heterogeneity makes recognition of patterns more complex and may result in convergence of deep learning models. After the application of feature engineering techniques, including rolling mean smoothing and lag-based feature generation, the resulting patterns (right panel of Fig. 4) became markedly smoother and more coherent. The three-hour rolling mean was introduced and increased the temporal consistency between the solar and wind production. This preprocessing refining enhances more robust model training in addition to the forecasting framework capacities to model seasonal and short-term dependencies in renewable energy generation.

**Fig. 5:**
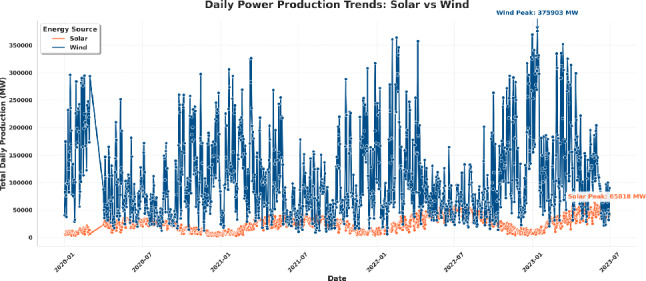
Daily power production trends for solar and wind energy sources over the observation period.

To better understand the temporal variability and long-term dynamics of renewable energy production, Fig. 5 illustrates the daily total power generation for solar and wind sources across the observation period. The visualization shows some obvious seasonal and inter-annual trends in both types of energy, which is indicative of the variability nature inherent to a meteorologically-dependent system.

As shown in Fig. 5, wind power exhibits substantially higher production levels and greater volatility than solar power, with several sharp peaks exceeding 350,000 MW, indicating its strong responsiveness to atmospheric fluctuations. On the contrary, solar production is a more gradual cyclical process determined by diurnal and seasonal irradiance, and its maximum peak is about 65, 818 MW. The fact that these two renewable types produce complementary energy highlights their ability to be integrated together in the energy grids, with wind energy compensating the times of low solar activity and the reverse. This visual comparison offers necessary information on intermittency in production, complementarity of resources and how supply can be maintained in systems that rely on renewables.

#### Normalization and encoding

To ensure numerical stability during model training, all continuous variables (Production, StartHour, EndHour, and dayOfYear) were normalized using a min–max scaling transformation. This scaling maps feature values into the range [0, 1] as defined by:

1 $$\begin{aligned} x_{\text {norm}} = \frac{x - x_{\min }}{x_{\max } - x_{\min }} \end{aligned}$$

Normalization ensures that all features contribute proportionally to the loss function, preventing gradient dominance by large-magnitude attributes and improving model convergence. Categorical variables, including Source, dayName, and monthName, were encoded numerically to enable compatibility with deep learning architectures. The Source variable was encoded using a binary scheme (0 for solar and 1 for wind), while multi-class variables such as dayName and monthName were transformed through one-hot encoding to avoid ordinal bias and preserve categorical independence.

**Fig. 6:**
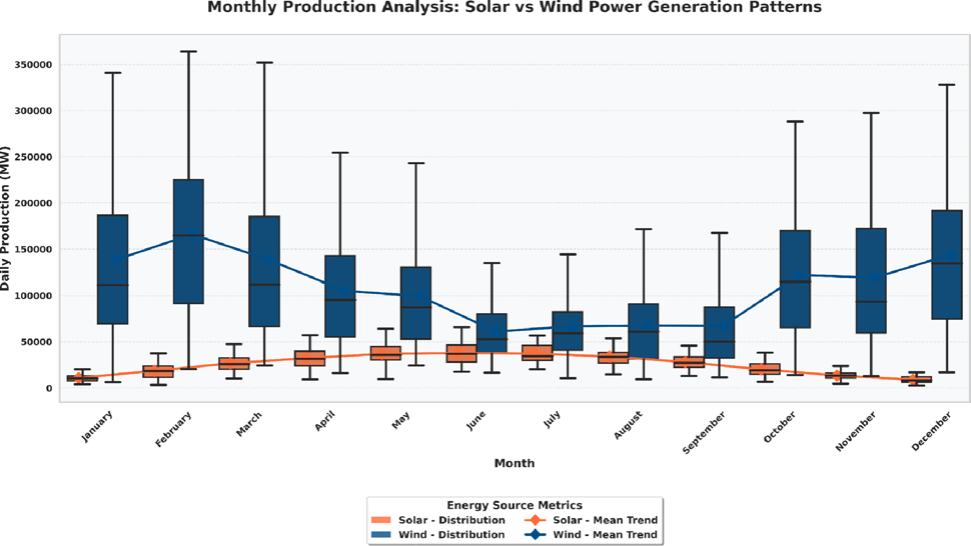
Monthly distribution and mean trends of daily solar and wind power production.

Following the completion of all preprocessing steps—validation, temporal structuring, interpolation, resampling, feature engineering, normalization, and encoding—the dataset was re-evaluated to confirm completeness and internal consistency. Statistical checks confirmed that the transformations preserved the original data distribution while enhancing its suitability for machine learning. The final preprocessed dataset was continuous, null-free, and standardized, providing a robust foundation for the development and optimization of the deep learning models discussed in the subsequent sections. The detailed study of the monthly pattern of the generation is important to determine the seasonal characteristics and complementary nature of solar and wind power sources. Figure 6 presents a detailed analysis of monthly production distributions for both energy sources, illustrating the variability and central tendency of their daily outputs throughout the year. This visualization helps in determining the cyclical patterns, the times of the year when the sources of the renewable energy give their best performances, and the degree of regularity that define each source.

As depicted in Fig. 6, wind energy production demonstrates substantial variability across all months, with wide interquartile ranges indicating strong dependence on meteorological fluctuations. Conversely, the generation of solar energy also has a much more predictable and seasonal trend in that it is highest during the summer months and decreases during the winter months. The mean trend lines indicate that there is a negative seasonal correlation between the two sources whereby the wind generated is usually higher in the colder months whereas the solar generated is higher in warmer months. This complementary trend emphasizes the synergistic opportunity of integrating solar and wind resource as a way of stabilizing the total renewable power production, as well as increasing grid reliability under different climatic conditions.

### Deep learning models

Renewable energy production prediction, especially solar and wind power production, is a data- and time-varying issue. The resulting complexity renders the application of models that can describe elaborate time-varying trends, taking into consideration the inherent variability of atmospheric dynamics, and respond to the nonlinear dependencies between the features. These tasks best fit the deep learning (DL) methods because of their ability to represent features in a hierarchical manner, scale to massive datasets, and adapt using optimization. In the case of the study, we look at a set of deep learning models that fulfill two complementary purposes. To begin with, we present a strong starting point, i.e., the Multi-Scale Attention Network (MSAN), a multi-resolution attention-based temporal forecasting method. Second, we consider a set of benchmark architectures that have shown resilient performance in such sequential prediction tasks in other contexts in terms of energy, healthcare and finance. Among them, one may distinguish between the Long Short-Term Memory (LSTM) network, the Gated Recurrent Unit (GRU), the Generative Adversarial Network on Time Series (GANT) and the Adaptive Residual Network (ARN).

**Multi-Scale Attention Network (MSAN)** The main model into which the forecasting framework of this study is built is the Multi-Scale Attention Network (MSAN). Its design is specific to pattern capture in multi-resolution temporal data, e.g., as in hourly, daily and seasonal variations in solar irradiance and wind flow. The algorithmic heart of MSAN is the idea that time-series data in the energy sector display natural time-hierarchies. As an example, the production of solar energy can exhibit readily predictable diurnal patterns, whereas wind patterns can exhibit seasonal patterns or sudden short-term variability. This is mitigated through MSAN, which breaks the input material into various sequences of time scales, through parallel encoding channels. The set of paths determines, per path, the dependencies to be captured at a given resolution. Individual encoders in MSAN scale-specific consist of the following:

- **Convolutional or Recurrent Layers:** These layers are responsible for local pattern extraction. Convolutional layers are used to learn spatially localized features in the temporal domain, while recurrent layers, particularly Gated Recurrent Units (GRUs), are used to preserve sequence order.
- **Multi-Head Attention Mechanisms:** Multiple attention heads operate in parallel to identify different positions in the input sequence that are contextually significant. The attention weights are learned during training and vary adaptively based on the input dynamics.
- **Fusion Layer:** The outputs of the multi-scale encoders are passed through a fusion layer where they are aggregated using a learnable weighting mechanism. This fusion ensures that the model attends to different temporal contexts as needed.
- **Residual Connections and Layer Normalization:** These are introduced to stabilize gradient propagation and improve convergence during training.
- **Dropout Regularization:** Dropout is applied to prevent overfitting by randomly deactivating neurons during training iterations.

Without any optimizations, MSAN achieves baseline accuracy in forecasting on both wind and solar data. Nonetheless, its architecture is flexible and complex, thereby allowing for extensive hyperparameter optimization and feature refinement, which will be selected to yield performance gains in models. Based on this, we deploy custom metaheuristic optimization an iHow Optimization Algorithm (iHOW)–to simultaneously optimize both architectural parameters and input feature selection strategy of MSAN. The iHOW-enhanced MSAN is the most advanced form in our comparison and shows greater modelling effectiveness, as indicated in subsequent sections. As a comparison, we assess a range of benchmark models that are generally considered as starting points in the literature concerning time series forecasting. Such models constitute various paradigms of architecture, including memory-augmented recurrent networks and adversarial encodings, such as generative and profound residual differences. Each model provides a different inductive bias, and the addition of them enables us to critically evaluate the relative merits of MSAN within identical evaluation protocols.

**Long Short-Term Memory Network (LSTM)** The Long Short-Term Memory network (LSTM) is a Recurrent Neural Network (RNN), designed by Hochreiter and Schmidhuber to overcome the vanishing gradient problem, using the memory cells and gates. Composed of only a single cell, the LSTM unit regularly retains an internal cell state that is controlled by three gates, such as the input gate, forget gate, and output gate. Such gates regulate the activity of information into and within the memory cell as well as out of it. A single LSTM cell can be mathematically formulated using the following as its formulation:

2 $$\begin{aligned} f_t&= \sigma (W_f \cdot [h_{t-1}, x_t] + b_f) \end{aligned}$$

3 $$\begin{aligned} i_t&= \sigma (W_i \cdot [h_{t-1}, x_t] + b_i) \end{aligned}$$

4 $$\begin{aligned} \tilde{C}_t&= \tanh (W_C \cdot [h_{t-1}, x_t] + b_C) \end{aligned}$$

5 $$\begin{aligned} C_t&= f_t * C_{t-1} + i_t * \tilde{C}_t \end{aligned}$$

6 $$\begin{aligned} o_t&= \sigma (W_o \cdot [h_{t-1}, x_t] + b_o) \end{aligned}$$

7 $$\begin{aligned} h_t&= o_t * \tanh (C_t) \end{aligned}$$

In this case, $f_t$, $i_t$, $o_t$ are forget, input and output gates respectively, $C_t$ cell state and $h_t$ hidden output. The structure has the advantage of allowing the LSTM to capture long-range dependencies and smooth temporal trends; thus, it could be of particular interest in applications that require long-range dependencies and smooth temporal trends, in particular, sun irradiance prediction and wind profile smoothing.

**Gated Recurrent Unit (GRU)** A simplified version of LSTM, the Gated Recurrent Unit (GRU) combines the gating process into two gates, the update gate and the reset gate. Even though it has simplified architecture, the GRU still has the temporal memory ability needed to perform sequential estimation tasks, but has lower computation complexity. GRUs are mostly preferred in real-time systems as well as real-world situations when the latency of model inference is a limitation. The GRU is easy to understand and serves as a good baseline in our study, making it easier to understand the trade-offs between the model’s depth, computational cost, and predictive ability.

**Generative Adversarial Network for Time Series (GANT)** GANT generalises the standard Generative Adversarial Network (GAN) paradigm to learn temporal structures in time-sequential data. It consists of two neural systems, including a generator and a discriminator, that are involved in a competitive learning process. The generator tries to generate realistic sequences with a condition on past observations, whereas the discriminator attempts to discriminate between real and fake sequences. Such an adversarial environment proves beneficial in learning data distributions that are complicated and possess a multimodal nature. GANT has demonstrated success in areas such as anomaly detection and sequence imputation. This study investigates the application of GANT in capturing uncertainty related to wind and solar production.

**Adaptive Residual Network (ARN)** The Adaptive Residual Network (ARN) extends a deep architecture to deep feedforward networks by inserting residual connections to reduce the training challenge that deep networks have faced. Residual blocks enable identity mapping, allowing gradients to flow freely across layers. In ARN, these connections are even reinforced with the adaptive weighting mechanism, which allows the model to expand/shrink residual pathways according to the input feature distributions. This flexibility makes ARN very effective in learning both stationary and non-stationary parts of data in time-series. This feature is essential in modeling abrupt weather-induced variability of renewable energy production. Our set of models is trained on a standardized training pipeline, which consists of a set of shared preprocessing routines, identical loss functions, and evaluation measures. This helps ensure that any difference in performance can be attributed solely to the model architecture and optimization knowledge, rather than experimental noise or preprocessing artefacts. MSAN model, especially as it would only be improved through the iHOW algorithm, would show competitive, possibly even better forecasting accuracy as compared to these benchmark models. However, it is critical to consider the existence of varied architectures independent of the experimental configuration to test the degree of model robustness, as well as to determine the circumstances where the alternative models can be superior to the baseline.

### Metaheuristic algorithms

Metaheuristic algorithms are a hybrid and multipurpose group of optimization methodologies that can be applied very well to finding solutions to high-dimensional, non-convex problems and complex optimization problems, of which the deep learning model development falls into. In contrast to gradient-based optimizers, which can be constrained by strict mathematical rules and may become stuck at local minima, metaheuristics incorporate stochastic elements inspired by natural processes, allowing for broader and looser exploration of the search space. Metaheuristics have been applied in the context of predictive modeling of renewable energy in two key functions: (i) feature selection, which combats the curse of dimensionality by representing a small set of informative variables; and (ii) hyperparameter tuning, which optimizes model designs to enhance predictive accuracy and generalization performance. The application of metaheuristics in such applications not only improves the performance of the model but also saves considerable time in development because it automates what would prove to be time-consuming and manual trial-and-error solutions.

#### Role in feature selection

Deep learning Feature selection is a crucial preprocessing step that reduces the dimensionality of data in computer-aided processes, where large amounts of high-dimensional data are generated. Unnecessary, meaningless, or even noisy characteristics can degrade the model’s performance, leading to overfitting, increased computational expense, and reduced interpretability. The best match to this challenge is the metaheuristic algorithms, as they operate well to search through large and discontinuous feature spaces. The elements of a metaheuristic algorithm are individual candidate solutions, all of which are binary encoded feature subsets, meaning that a candidate solution has a value of either 1 to include a feature or 0 to exclude it. The problem of selection can be performed automatic depending on the objective function that is usually expressed as model accuracy or a composite measure of accuracy and model size. This process of optimization is an iterative process where populations of candidate solutions change over time according to the principles of the underlying algorithmology, whether that is swarm intelligence, evolutionary strategy or physics-inspired dynamics. An example of such methods is the algorithms Binary Grey Wolf Optimizer (bGWO), Binary Particle Swarm Optimization (bPSO), and Binary Firefly Algorithm (bFA) included in the list of abbreviations in the document. These algorithms can accommodate exploration and exploitation to process earlier convergence and instead progress incrementally through an optimal or near-optimal feature set. The main advantages of metaheuristic-based FS are twofold: (1) the dimension of the input is reduced, and that verifies in shorter training and inference times, and (2) the noise and collinearity between features are removed; after all, this will enhance predictive stability and robustness of the outputting models.

#### Role in hyperparameter optimization

Whereas the architecture of a deep learning model establishes the structure of learning, the choice of hyperparameters—learning rate, dropout rate, the number of hidden layers, the number of attention heads, batch size, and weight decay—determines the dynamics of training and the ultimate generalization capacity. Slow convergence, overfitting, underfitting, and even training failure may be the results of the suboptimal hyperparameter choice. Therefore, the process of hyperparameter optimization is an essential element of the development of a model, sometimes more significant than architectural decisions themselves. Manual tuning using domain-based heuristics and commonly used grid search are traditional methods of doing this task. Nonetheless, this type of approach is computationally demanding, inflexible and non-scalable to large search spaces. A lovely solution exists in metaheuristic algorithms that view the issue of hyperparameter tuning as a global optimization problem. Within this framework, every candidate solution is a real-valued vector that represents a particular configuration of hyperparameters. Each solution is evaluated by training the model to utilize the parameters and assessing performance on a single validation set and a single training set, using metrics such as Mean Squared Error (MSE), Root Mean Squared Error (RMSE), or $R^2$. Grey Wolf Optimizer (GWO), Particle Swarm Optimizer (PSO), Biogeography-Based Optimizer (BBO) and researcher-proposed iHow Optimization Algorithm (iHOW) are among such algorithms that are effective in such settings owing to the learning mechanisms of searches and convergence of the algorithms. The above approaches are based on the dynamic adaptation of candidate solutions to the fitness landscape of the population so that global exploration and local exploitation are balanced. Moreover, the combination of metaheuristic optimization and cross-validation allows for guaranteeing that the chosen hyperparameters will be generalizable sufficiently over different subsets of data. Hence, the model improves its stability. The use of metaheuristics allows reducing the reliance on human guesswork and making the hyperparameter search data-driven and automated, efficient and practical at the same time. The use of metaheuristic algorithms in the design of features to work in committee and to tune the hyperparameters provides a synergetic benefit. Combined, these techniques result in not only computationally efficient but also very predictive and robust against overlearning forecasting models. They are an essential part of the modeling pipeline that is used in the present research and are further discussed in Sect. 3.4, where particular algorithms and details of their implementation are presented.

#### Proposed optimizer: ihow optimization algorithm

According to the given study, the proposed metaheuristic approach, a new and recently published *iHow Optimization Algorithm (iHow)*, is used to optimize both feature selection and hyperparameter tuning. Contrary to classical population-based methods, which are based on the behaviour of animals, physical systems, or evolutionary processes, iHow has its conceptual foundation based on the processes of human cognition, i.e., *learning, information acquisition, decision-making, and expertise acquisition*. This particular inspiration makes iHow a cognitively competitive algorithm that is meant to solve high-dimensional, nonlinear and multimodal optimization problems. The main novelty in iHow is its ability to simulate the adaptive learning strategies of human beings as they progress from novices to experts. The algorithm is structured into a five-tier knowledge hierarchy, which resembles human development overall: raw Data gathering, followed by Learning, Information processing, and Knowledge accumulation, and eventually culminates in expertise, a process that altogether concrizes intelligent refinement of solutions as time goes by. Every phase builds in feedback and heuristic controls, which enable an exploration/exploitation tradeoff between the solution space and the high-fitness regions. This search behavior in iHow is controlled through a collection of dynamic update equations with learning rates and a temporal knowledge factor. This amount of knowledge, represented by K, decreases throughout the iterations to enhance the transition of focus from exploration to exploitation^42^. The algorithm may be mathematically divided into the following stages:

- **Exploration Phase:** Candidate solutions are generated within the search space to foster diversity. Updates in position involve weights based on combinations of stochastic factors, similar to the initial phases of collecting information.
- **Learning Phase:** Solutions start changing after early responses. The revision is controlled by the accumulated information after processing the terms whose rates are modulated by the learning rate.
- **Knowledge Acquisition:** Patterns applied through learning are stored in a knowledge base, which affects the subsequent generation of candidates. The changing state of knowledge balances the effects between exploration and exploitation.
- **Exploitation Phase:** Candidate solutions concentrate searches in promising regions discovered through a build-up of experience. Precision is increased by local, knowledgeable adjustments based on feedback.
- **Best Solution Synthesis:** The impact of exploration vectors, learning updates and knowledge states is combined in a composite formula that defines the best solution at the iteration.

All these stages are mathematically reflected in the algorithmic procedure, meaning that complex mathematical models are present as well as an update procedure that is coupled with a convergence control. The iterative structure of the pseudo-code, which starts with population initialization and termination, is also clearly articulated in the pseudo-code provided in Algorithm 1.

** Algorithm 1** Proposed iHow Optimization Algorithm (iHow)

**Figure:**
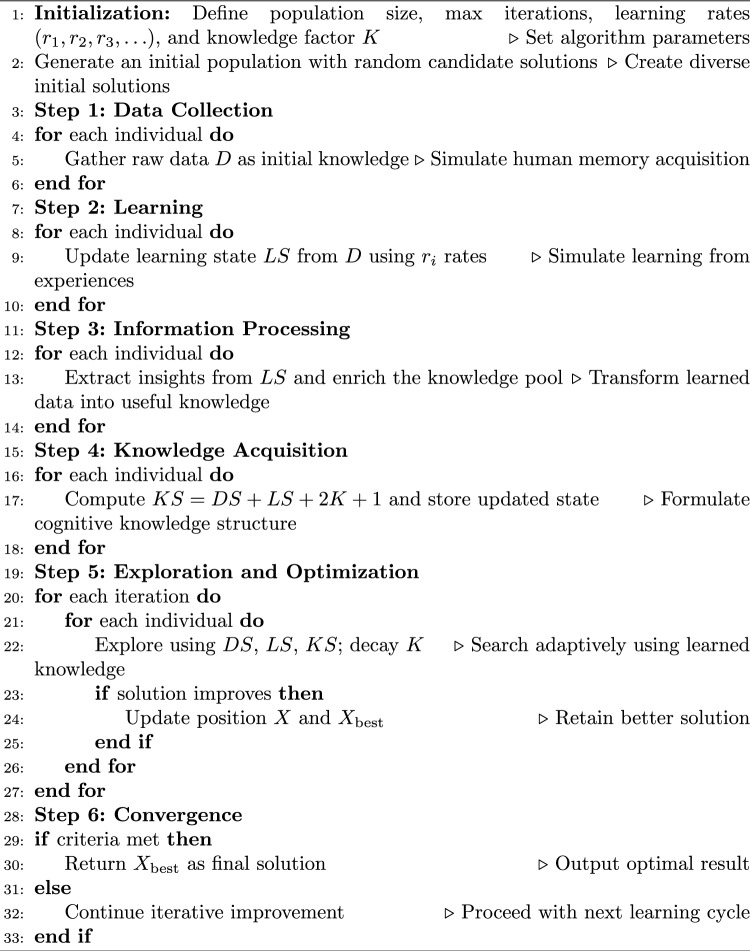

The iHow was designed not merely as a generic optimizer but as a domain-adaptive tool capable of high precision in tasks such as feature selection and hyperparameter tuning—two of the most computationally demanding components in deep learning.

- For **feature selection**, iHow incorporates a simple binary adaptation (biHow) to select the most pertinent subset of input features, dramatically decreasing data dimensionality in a way that does not affect the accuracy of classification of the data. The binary version employs a discretization process to encode continuous locations as binary inclusion vectors.
- For **hyperparameter optimization**, iHow can efficiently find the maximum learning rates, the heads-per-attention size, dropout rates, number of encoder layers, and batch sizes in a continuous parameter space. Its self-adaptive knowledge-based architecture does not require thorough grid search procedures or parameter settings made by hand.

Experimental works provided in ^43^ have shown that iHow performs better than fully developed optimizers, including Harris Hawks Optimization (HHO), Particle Swarm Optimization (PSO), Differential Evolution (DE), and Sine-Cosine Algorithm (SCA), on real-world tasks and benchmark functions (CEC 2005 suite). Particularly, the project iHow implemented:

- **Zero mean and standard deviation** of convergence accuracy of various unimodal and multimodal test functions.
- **Reduced computational time** With no increased number of function evaluations.
- **Smaller selected feature sets** And reduces classification error, in binary feature selection benchmarks, relative to bHHO, bDE and bSCA.

These outcomes confirm the strength, scalability, and general viability of iHow in complicated optimization terrain. Considering the human-centric origin, mathematical basis of the search process, and success in high-stakes, large-scale benchmarks, the iHow Optimization Algorithm would declare an interesting new contribution to metaheuristic optimization. Its doubly effective feature selection and hyperparameter optimization ability render it central to the framework of the optimization process proposed in this paper to make intelligent predictions of solar and wind energy generation.

#### Benchmark optimizers for comparison

To critically evaluate the performance of the proposed ** iHow Optimization Algorithm (iHow)** in view of other popular existent well-tested algorithms in metaheuristic literature, we present a comparative study of the proposed algorithm with a range (suite) of such algorithms. These benchmark algorithms were diligently selected based on being broadly used and theoretically and experimentally successful on a broad range of optimization tasks, such as feature selection, hyperparameter tuning, time-series prediction and energy system optimization. All of these optimizers were deployed in two settings: (1) in a binary encoding format to address the feature selection problem, where the bits of the bitstring indicate the participation or non-participation of each of the individual features; and (2) in a continuous encoding format to support the hyperparameter optimization problem, where the candidate solutions represent continuous-valued parameters in the configuration of the deep learning models. The following are optimizers better known in the benchmarking of this study:

- **Harris Hawks Optimization (HHO):** HHO is based on the following inspirations found in the predatory behavior of the Harris Hawks: surprise pounce strategies, a series of four dynamic phases to capture prey, and modeling exploration and exploitation based on prey escape energy and movement strategy. It is a good baseline since it succeeds in non-linear and multimodal problems^44^.
- **Grey Wolf Optimizer (GWO):** GWO is modeled after the leadership structure and hunting strategies of grey wolves, which depict social dominance and tracking, encircling, and attacking behaviors. It enjoys a rapid convergence rate and robust global search power^45^.
- **Particle Swarm Optimization (PSO):** A social optimization algorithm, imitating the flocking phenomena of birds and fish. PSO remembers the best solution, either locally or globally and uses this information to enable the adjustment of the trajectory of particles exploring the solution space toward regions that are promising^46^.
- **Whale Optimization Algorithm (WAO):** WAO mimics the ability of the humpback whales to break up a food source with their bubble-net feeding method. It is based on the technique of surrounding prey and updating positions in spirals, which is favourable to gradient-free optimization problems overall^47^.
- **Biogeography-Based Optimizer (BBO):** This algorithm generates ideas based on the geographical distribution of species. Migration, emigration rates and habitat suitability index determine the exchange of information among solutions^48^.
- **Multi-Verse Optimizer (MVO):** MVO is based on cosmological theories to inform the population about functional solutions, utilizing concepts of black holes, white holes, and wormholes to maintain diversity^49^.
- **Stochastic Fractal Search (SFS):** Requires a fractal exploration strategy that uses random walks and step size variations to move effectively across the solution landscape^50^.
- **Simulated Annealing Optimization (SAO):** SAO was introduced as a process inspired by the physical annealing process in metallurgy. It allows for probabilistic transitions to worse solutions, enabling escape from local minima, and achieves a trade-off between exploration and exploitation by controlling temperature-related convergence^51^.
- **JAYA Algorithm:** JAYA Algorithm is a general goal-oriented optimizer, which is a parameter-free optimizer algorithm, and always tries to move in the direction of the best solution and the direction of the worst solution with no need for a control coefficient specific to the algorithm. When operating under a limited situation, its simplicity and the reliability of its convergence become an appealing feature^52^.

Parameters recommended by the literature were used in all algorithms. All algorithms run on identical experimental settings, on the same datasets and using the same measures of fairness and reproducibility. The empirical findings of the benchmark approaches serve as a reference for evaluating the accuracy, convergence speed, and computational efficiency of iHow in both forecasting and feature selection processes.

### Evaluation metrics

To thoroughly and effortlessly assess the merits of both deep learning prediction models and the performance of the metaheuristic optimizers, a range of quantitative tests was employed. Those numbers cover both error-based forecasting measures and optimization-focused metrics of feature selection effectiveness.

#### Forecasting performance metrics

The measures used to forecast gauge how well the predictive models perform given the selected features and optimized hyperparameters, as reported in Table  3 .Such measures assess the consistency between the estimated values $\hat{y}_i$ and the actual values $y_i$ in several statistical ways - absolute error, squared error, bias, correlation, and explained variance.

**Table 3:** Forecasting performance metrics and their mathematical definitions.

Metric	**Mathematical Definition**
Mean Squared Error (MSE)	$\displaystyle \text {MSE} = \frac{1}{n} \sum _{i=1}^{n} (y_i - \hat{y}_i)^2$
Root Mean Squared Error (RMSE)	$\displaystyle \text {RMSE} = \sqrt{ \frac{1}{n} \sum _{i=1}^{n} (y_i - \hat{y}_i)^2 }$
Mean Absolute Error (MAE)	$\displaystyle \text {MAE} = \frac{1}{n} \sum _{i=1}^{n} |y_i - \hat{y}_i|$
Mean Bias Error (MBE)	$\displaystyle \text {MBE} = \frac{1}{n} \sum _{i=1}^{n} (y_i - \hat{y}_i)$
Pearson Correlation Coefficient (r)	$\displaystyle r = \frac{ \sum _{i=1}^{n} (y_i - \bar{y})(\hat{y}_i - \bar{\hat{y}}) }{ \sqrt{ \sum _{i=1}^{n} (y_i - \bar{y})^2 } \sqrt{ \sum _{i=1}^{n} (\hat{y}_i - \bar{\hat{y}})^2 } }$
Coefficient of Determination ($R^2$)	$\displaystyle R^2 = 1 - \frac{ \sum _{i=1}^{n} (y_i - \hat{y}_i)^2 }{ \sum _{i=1}^{n} (y_i - \bar{y})^2 }$
Relative RMSE (RRMSE)	$\displaystyle \text {RRMSE} = \frac{\text {RMSE}}{\bar{y}} \times 100$
Nash–Sutcliffe Efficiency (NSE)	$\displaystyle \text {NSE} = 1 - \frac{ \sum _{i=1}^{n} (y_i - \hat{y}_i)^2 }{ \sum _{i=1}^{n} (y_i - \bar{y})^2 }$
Willmott Index (WI)	$\displaystyle \text {WI} = 1 - \frac{ \sum _{i=1}^{n} (y_i - \hat{y}_i)^2 }{ \sum _{i=1}^{n} ( |y_i - \bar{y}| + |\hat{y}_i - \bar{y}| )^2 }$

These metrics of forecasting give a representative perception of the quality of the predictive models. MSE, RMSE and MAE are estimates of the magnitude of the error; MBE is an estimate of directional bias; r and squared *r* are the measurements of correlation and the explanation of variance; RRMSE, NSE, and WI are normalized evaluations, depending on the context.

#### Feature selection performance metrics

To analyse the goodness and stability of the feature subsets generated by each metaheuristic algorithm, the following metrics related to the optimization problem were involved, as summarized in Table  4 :

**Table 4:** Feature selection evaluation metrics and their formulations.

Metric	Mathematical Definition
Average Classification Error (AE)	$\displaystyle \text {AE} = \frac{1}{M} \sum _{j=1}^{M} \frac{1}{N} \sum _{i=1}^{N} \text {mse}(V_i - \hat{V}_i)$
Average Selected Feature Size	$\displaystyle \text {AvgSize} = \frac{1}{M} \sum _{i=1}^{M} |\mathcal {S}_i|$
Average Fitness Score	$\displaystyle \text {AvgFitness} = \frac{1}{M} \sum _{i=1}^{M} F_i$
Best Fitness Score	$\displaystyle \text {BestFitness} = \min _{i \in [1,M]} F_i$
Worst Fitness Score	$\displaystyle \text {WorstFitness} = \max _{i \in [1,M]} F_i$
Standard Deviation of Fitness	$\displaystyle \text {StDev} = \sqrt{ \frac{1}{M - 1} \sum _{i=1}^{M} (F_i - \bar{F})^2 }$

Taken together, these metrics evaluate the trade-off between classification accuracy and dimensionality reduction. Both algorithmic strength and solution strength are reflected in high fitness values, smaller feature subsets, and slight standard deviation among runs of an optimization.

## Experimental setup

This section presents the experimental configuration used to train and evaluate the Multi-Scale Attention Network (MSAN) optimized by the iHow Optimization Algorithm (iHOW). The configuration includes details of the model architecture, attention design, optimization and training parameters, and computational hardware. This structured setup ensures reproducibility and supports an accurate comparison with existing baselines.

### Model architecture and training parameters

The suggested forecasting model is built on the Multi-Scale Attention Network (MSAN), which focuses on both short and long-term dependencies in the data of renewable energy. Table below, Table 5 , in short summarises the architectural and attention parameters of the MSAN which combine multi-scale temporal processing with multi-head attention mechanisms.

**Table 5:** MSAN Model Architecture and Attention Parameters.

Parameter	Value/Description
*Architecture Parameters*	
Model Type	Multi-Scale Attention Network (MSAN)
Number of Layers	8
Hidden Units	512
Embedding Dimension	256
Feedforward Dimension	1024
Scale Levels	4
*Attention Mechanism*	
Multi-Head Attention	Enabled
Number of Heads	8
Attention Dimension	64
Attention Dropout	0.1
Causal Attention	Disabled

The hierarchical attention and multiscale temporal representation of the MSAN architecture successfully capture nonlinear relationships within the data of the solar and wind production systems. This information is robust and interpretable because it considers high-frequency and low-frequency temporal features.

### Optimization and training configuration

AdamW optimizer was used to optimize the training process because it has an adaptive learning property, which is effective in regularization. Cosine annealing schedule was utilized to smooth the learning rate and gradient clipping was utilized to stabilize updates. The general training parameters will be listed in The following Table 6.

**Table 6:** Optimization and Training Parameters.

Parameter	Value/Description
*Optimization Settings*	
Optimizer	AdamW
Learning Rate	0.001
Weight Decay	0.01
Beta1/Beta2	0.9/0.999
Epsilon	$1\times 10^{-8}$
Gradient Clipping	1.0
Learning Rate Schedule	Cosine Annealing
*Training Settings*	
Batch Size	64
Epochs	500
Validation Split	0.2
Early Stopping Patience	30 epochs
Shuffle	Enabled
Random Seed	42

These configurations ensure efficient model convergence while mitigating overfitting. Early stopping with patience prevents excessive training beyond convergence, and shuffling maintains randomness across epochs for improved generalization.

### Hardware configuration

All experiments were executed on a dedicated high-performance workstation optimized for deep learning workloads. The system configuration is summarized in The following Table 7.

**Table 7:** Hardware and System Configuration.

Component	Specification
CPU	Intel Core i9-14900K (8P + 16E cores, 5.8 GHz Turbo)
Motherboard	ASUS ROG Maximus Z790 Hero
RAM	64 GB G.Skill Trident Z5 RGB DDR5 (6000 MHz, CL30)
GPU	NVIDIA GeForce RTX 4090 (24 GB GDDR6X)
Primary Storage	2 TB Samsung 990 Pro NVMe PCIe 4.0 SSD
Secondary Storage	4 TB WD Black SN850X NVMe PCIe 4.0 SSD
PSU	Corsair HX1200 1200W 80+ Platinum
Cooling System	Arctic Liquid Freezer II 420 mm AIO
Chassis	Lian Li Lancool III RGB

This configuration ensured stable performance, rapid computation, and sufficient memory bandwidth for training deep architectures. The use of an RTX 4090 GPU substantially accelerated attention computations and enabled efficient experimentation with large-scale temporal datasets.

## Empirical results

In this section, the empirical findings discussed in the previous sections are synthesized, resulting in a critical interpretation of the results on forecasting performance and optimization. It aims to test the advantages and weaknesses of the suggested hybrid technique, i.e., the combination of Multi-Scale Attention Network (MSAN) with the Binary iHow Optimization Algorithm (biHOW), against models of deep learning-based baselines and traditional metaheuristics. Through a deliberate discourse on the accuracy of the forecast, the feature selection aspect of efficiency, and the hyperparameter optimization performance, we aim to highlight the major contributors to the model’s performance gain and the broader implications of intelligent renewable energy forecasting. This also includes the reflection of the discussion on how generalizable the proposed methodology is, and the way to promote improvement in adaptive energy systems in the future.

### Baseline deep learning performance (before feature selection)

The advantages of feature selection and optimization required a reference point; thus, a comparative analysis was conducted first with the unoptimized versions of all deep learning models to determine this. They are the Multi-Scale Attention Network (MSAN), Long Short-Term Memory network (LSTM), Gated Recurrent Unit (GRU), Generative Adversarial Network for Time Series (GANT) and Adaptive Residual Network (ARN). Each of the models was trained and tested using the same wind forecasting dataset, the same data splits, and the same data preprocessing processes. The **MSAN architecture performed best in baseline forecasting performance** in almost all measures of evaluation. In particular, MSAN achieved optimal results, i.e., coefficient of determination ( $R^2 = 0.8558$), correlation coefficient ($r = 0.8432$), and Nash-Sutcliffe efficiency (NSE = 0.8638). These values signify good accuracy and model reliability in terms of capturing the temporal dynamics of wind energy production, even before any optimization techniques have been applied so far. Table 8 gives an overview of the results of all models, comparing them. The metrics use some of the traditional forecasting metrics Mean Squared Error (MSE), Root Mean Squared Error (RMSE), Mean Absolute Error (MAE), Mean Bias Error (MBE) and correlation (*r*, $R^2$, RRMSE, NSE, and Willmott index (WI)).

**Table 8:** Baseline forecasting performance of deep learning models before feature selection (wind forecasting).

Model	MSE	RMSE	MAE	MBE	$\boldsymbol{r}$	$\boldsymbol{R^2}$	RRMSE	NSE	WI
MSAN	0.0105	0.0055	0.0054	0.0051	0.8432	0.8558	23.49	0.8638	0.8592
LSTM	0.1226	0.0543	0.0504	0.0788	0.8033	0.8159	25.70	0.8546	0.7399
GRU	0.3290	0.0599	0.0554	0.0876	0.6960	0.7086	26.53	0.8017	0.6840
GANT	0.7110	0.1348	0.1241	0.1857	0.6597	0.6723	27.11	0.7776	0.6200
ARN	0.8443	0.1563	0.1443	0.9258	0.6412	0.6537	27.54	0.7517	0.5536

**Fig. 7:**
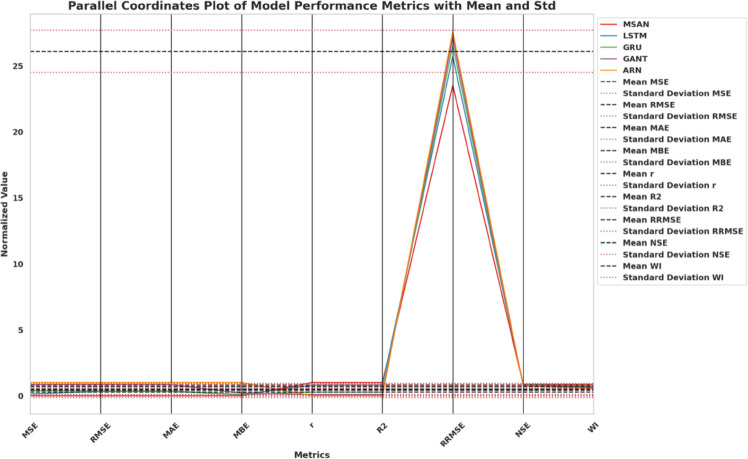
Parallel coordinates plot comparing the normalized performance metrics of different wind forecasting models along with their corresponding mean and standard deviation values.

The substantially reduced error measurements and the greater explanatory scope of the MSAN mode, considering the rest of the architectures, reveal its capacity to mine multi-scale extension of the temporal patterns of the wind data. In the meantime, the conventional RNN-based solutions (e.g., LSTM and GRU) have shown solid results. However, they have had a larger forecasting error and featured a slight drop in the generalization power. GANT and ARN, which are more loosely structured, performed worse on all measures, particularly in terms of bias and variance explanation. The obtained baseline results provide a solid rationale for using feature selection and hyperparameter optimization methods in the further implementation of the research process, aiming to improve predictive performance and model robustness even further. Wind energy potential should be correctly predicted to integrate renewable resources into the power grid effectively. Many postulated models of machine learning and deep learning aim at increasing the precision of the prediction of wind speed. To adequately assess the efficiency of such models, we resort to a comprehensive evaluation of them based on various statistical quantifiers. Figure 7 presents the parallel coordinates diagram of the normalized model results of some of the models containing MSAN, LSTM, GRU, GANT, and ARN. The measured criteria, e.g., MSE, RMSE, MAE, MBE, the correlation coefficient (*r*), coefficient of determination ($R^2$), RRMSE, NSE, and WI, account for multiple dimensions of performance of the forecasting accuracy. Also, the figure provides the mean and standard deviation of each of the metrics, providing an idea of how consistently and robustly the models perform regarding all the criteria of evaluation.

The approach adopted to determine the performance of the model under unoptimized conditions of solar forecasting is to apply the same baseline architectures to the dataset of solar power production. These are the Multi-Scale Attention Network (MSAN), Long Short-Term Memory network (LSTM), Gated Recurrent Unit (GRU), Generative Adversarial Network for Time Series (GANT) and Adaptive Residual Network (ARN). The exact amount of data was used during the training of model models, whereas data splits and preprocessing were also done identically to make comparisons fair and comparable. According to the findings summarized in Table 9, the **MSAN model has the best forecasting results in most of the metrics** even without feature selection or hyperparameter tuning. In particular, MSAN produced a higher value of $R^2$ (0.8299), Pearson correlation coefficient r (0.8173), Nash-Sutcliffe efficiency (NSE) (0.8480), and Willmott index (WI) (0.8433) as compared to other models. These measures consistently demonstrate the superiority of MSAN in reproducing the non-linear and periodic character of solar irradiance data.

**Table 9:** Baseline forecasting performance of deep learning models before feature selection (solar forecasting).

Model	MSE	RMSE	MAE	MBE	$\boldsymbol{r}$	$\boldsymbol{R^2}$	RRMSE	NSE	WI
MSAN	0.0976	0.0508	0.0501	0.0474	0.8173	0.8299	22.48	0.8480	0.8433
LSTM	0.2677	0.1186	0.1102	0.1720	0.7774	0.7900	24.68	0.8387	0.7240
GRU	0.7186	0.1308	0.1209	0.1914	0.6701	0.6827	25.52	0.7858	0.6681
GANT	0.7539	0.1429	0.1316	0.1969	0.6338	0.6464	26.10	0.7617	0.6041
ARN	0.7927	0.1468	0.1355	0.9816	0.6153	0.6278	26.53	0.7358	0.5377

**Fig. 8:**
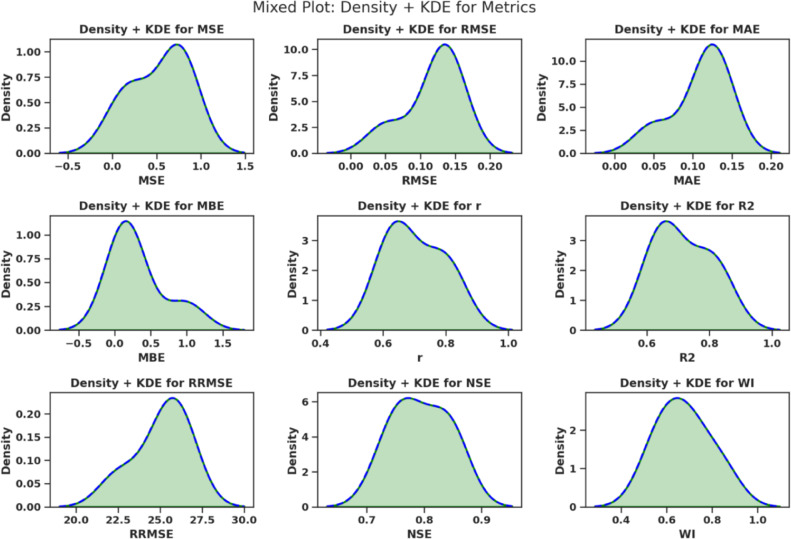
Density and KDE plots for evaluation metrics in solar energy forecasting. These plots illustrate the probabilistic distribution of key performance indicators used to assess model effectiveness.

Not only does data show MSAN has the lowest error rates (MSE = 0.0976, MAE = 0.0501), but it is also the most consistent in its predictions as indicated by maximum values of $R^2$, NSE, and WI (as evident in Table 9). These findings are as anticipated since the multi-scale attention modules in MSAN make it adaptable to both the short-term variability and the long-term trends that can be seen in solar irradiance data. Conversely, ARN and GANT models all performed worse when compared to other methods in all the metrics. ARN did record the strongest error in forecasting (MSE = 0.7927), weakest $R^2 = 0.6278$ and unstable NSE = 0.7358. The GANT model was not very successful either in achieving a better result than historical methods based on RNN models, which indicates potentially inappropriate application of adversarial generation in a deterministic forecasting task of solar power data. These findings ensure that MSAN provides the best baseline architecture for solar forecasting applications. Nevertheless, the exceptionally high performance gap between MSAN and the rest also marks the potential usefulness of greater optimization not only in input dimensionality-reduction on a feature-selection basis but also in better convergence via hyperparameter optimization, consequently. These are the aspects discussed in the following sections. During solar energy prediction, the distribution features of the evaluation measure are essential for diagnosing the model’s behavior and predicting its reliability. Probabilistic visualizations, such as density plots and kernel density estimation (KDE), help understand the spread, skewness, and modality of metrical distributions. Figure 8 shows all of the significant performance indicator plots such as MSE, RMSE, MAE, MBE, correlation coefficient (*r*), coefficient of determination ($R^2$ ), RRMSE, NSE and WI. With these plots, one can experience a subtle analysis of model performance in estimating solar radiation as the concentration of points across the measures could be diagnosed to suggest the presence of outliers.

### Feature selection results

Feature selection is a primary method used to reduce the dimensionality of the input data to maintain model performance or even enhance its performance. This paper compares ten binary metaheuristic algorithms in terms of their aptitude to choose the most applicable features: (biHOW), (bHHO), (bGWO), (bPSO), (bWAO), (bBBO), (bMVO), (bSFS), (bSAO), and (bJAYA). The evaluation of the performance of every optimizer was done in the following ways: an average classification error, average number of chosen features (as a percentage of the total features), average fitness, lowest and optimum fitness in every trial, and the standard deviation of the fitness score. Table 10 shows the results of wind and Table 11 shows the results of solar implementations. Both data sets indicated that the proposed biHOW algorithm generated the minimal mean classification error along with the minimal mean selected feature set, demonstrating that the minimal-sized set of informative features can be identified. These findings bear out the efficient work of biHOW that keeps the balance between accuracy in classification and dimensionality reduction.

**Table 10:** Feature selection results on wind dataset using binary metaheuristics.

Optimizer	Avg. Error	Avg. Size	Avg. Fitness	Best Fitness	Worst Fitness	Std. Dev.
biHOW	**0.3925**	**0.3453**	**0.4557**	**0.3575**	**0.4560**	**0.2780**
bHHO	0.4102	0.5458	0.4724	0.3927	0.4596	0.2832
bGWO	0.4495	0.6791	0.4807	0.4342	0.5442	0.3014
bPSO	0.5430	0.6448	0.5698	0.5501	0.6178	0.3816
bWAO	0.5428	0.8082	0.5776	0.5417	0.6178	0.3838
bBBO	0.5112	0.8086	0.5755	0.5652	0.6517	0.4265
bMVO	0.5197	0.7413	0.5995	0.5247	0.6427	0.4323
bSFS	0.4126	0.5482	0.4748	0.3951	0.4620	0.2856
bSAO	0.4560	0.6876	0.4961	0.3858	0.4874	0.2949
bJAYA	0.4462	0.7116	0.4810	0.4451	0.5212	0.2872

**Fig. 9:**
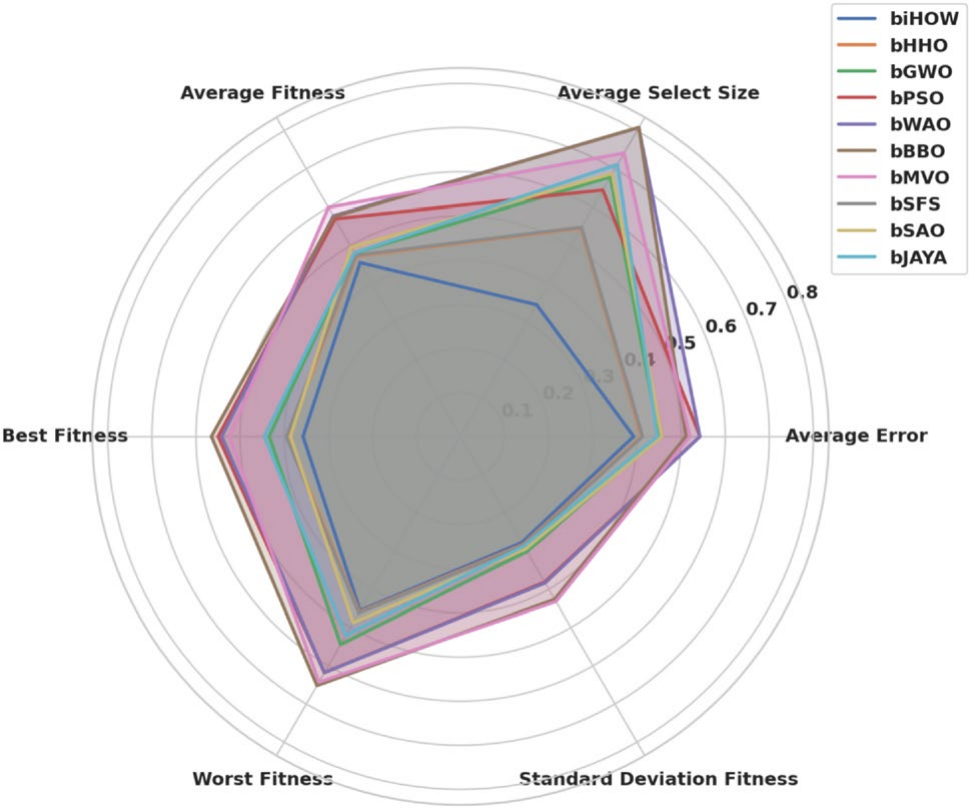
Radar plot comparing the performance of various bio-inspired optimization algorithms across multiple fitness and error-based metrics in wind energy forecasting.

As Table 10 reveals, biHOW exhibited the lowest average classification error (0.3925), the minimal feature subset (0.3453), and the highest average fitness score (0.4557) and nevertheless managed to have the lowest worst fitness (0.4560) and standard deviation (0.2780). Such outcomes point to excellent stability of convergence and stable optimization. To enhance the predictive accuracy of wind energy forecast models and optimize their performance, the use of commendable optimization algorithms in training and feature selection is crucial. A mixed collection of bio-inspired and evolutionary algorithms was also benchmarked on a set of evaluation parameters, including mean error, mean select size, mean fitness, best fitness, worst fitness, and the standard deviation of the fitness value. All these measures give a multidimensional perspective on the capability of every algorithm to search the search space and come to the best solutions. The comparative radar graph illustrates these optimization strategies and their pros and cons about wind energy modeling in Fig. 9.

Table 11 affirms that biHOW maintained its superiority on all metrics with solar data. It had the least mean error (0.4161), the least feature subset (0.3689), and the highest global fitness (0.4793), and its convergence characteristics were also steady, as illustrated by the least standard deviation of all algorithms. The biHOW outperforming feature in solar and wind experiments consistently illustrates robust generalization tendencies and flexibility to all realms of forecasting. Its feature dimensionality reduction capability, coupled with the ability to maintain predictive accuracy, substantiates its meaning of being a reliable and intelligent feature selection mechanism in the proposed forecasting platform.

**Table 11:** Feature selection results on solar dataset using binary metaheuristics.

Optimizer	Avg. Error	Avg. Size	Avg. Fitness	Best Fitness	Worst Fitness	Std. Dev.
biHOW	**0.4161**	**0.3689**	**0.4793**	**0.3811**	**0.4796**	**0.3016**
bHHO	0.4339	0.5695	0.4961	0.4164	0.4833	0.3069
bGWO	0.4732	0.7028	0.5044	0.4579	0.5679	0.3251
bPSO	0.5667	0.6685	0.5935	0.5738	0.6415	0.4053
bWAO	0.5665	0.8319	0.6013	0.5654	0.6415	0.4075
bBBO	0.5349	0.8323	0.5992	0.5889	0.6754	0.4502
bMVO	0.5434	0.7650	0.6232	0.5484	0.6664	0.4560
bSFS	0.4363	0.5719	0.4985	0.4188	0.4857	0.3093
bSAO	0.4797	0.7113	0.5198	0.4095	0.5111	0.3186
bJAYA	0.4699	0.7353	0.5047	0.4688	0.5449	0.3109

**Fig. 10:**
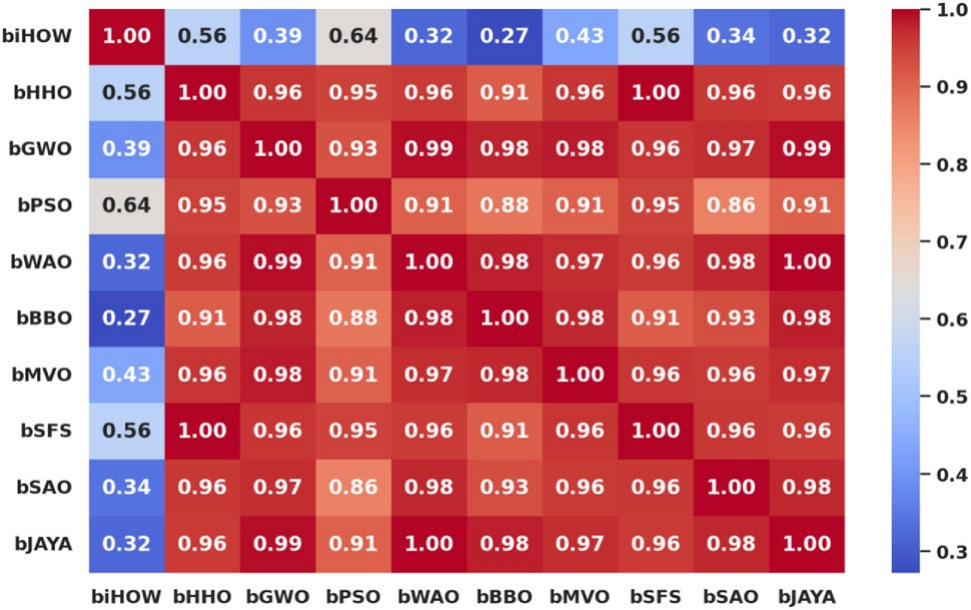
Correlation heatmap showing the pairwise similarity of bio-inspired optimization algorithms used in solar forecasting based on their performance metrics.

Comparing various optimization algorithms for predicting solar energy can help discuss how they behave similarly in terms of optimization. It is possible to use correction analysis to determine the redundant or complementary model patterns, which in turn stimulate the selection and ensemble methods. The Fig. 10 shows a Pearson correlation heatmap to indicate the quantitative measure of the relationship of pairs of various bio-inspired optimizers in terms of their profile in performances. The intensity of the color and the annotated values represent the extent of linear association, and the higher the value, the stronger the similarity in the behavior across a metric or a trial. The visualization provides insight into which strategies yield similar results and which represent distinct modeling properties.

### Forecasting results after feature selection

Following the implementation of feature selection methodologies, particularly through the binary version of the iHow Optimization Algorithm (biHOW), a significant improvement was observed in all performance scorecards of every deep learning model. The dimensionality reduction of the input data not only circumvented the computational challenge but also increased the predictive generalization. These improvements are especially significant in the MSAN model, where it produced the lowest forecasting errors in the wind and solar prediction tasks, and the highest statistical accuracy in both. The parameters/metrics applied in the given section agree to those that were presented at the beginning, such as Mean Squared Error (MSE), Root Mean Squared Error (RMSE), Mean Absolute Error (MAE), Mean Bias Error (MBE), Pearson correlation coefficient, (*r*), Coefficient of Determination, ($R^2$), Relative RMSE (RRMSE), NashSutcliffe Efficiency (NSE), and Willmott Index (WI). Tables 12 and 13 present a summary of the results of the post-selection forecasting used to study the education foundations research.

**Table 12:** Forecasting performance of deep learning models after feature selection (wind forecasting).

Model	MSE	RMSE	MAE	MBE	$\boldsymbol{r}$	$\boldsymbol{R^2}$	RRMSE	NSE	WI
MSAN	**0.0018**	**0.0007**	**0.0007**	**0.0006**	**0.8916**	**0.9042**	**15.44**	**0.8906**	**0.8860**
LSTM	0.0275	0.0108	0.0103	0.0168	0.8518	0.8644	17.64	0.8814	0.7825
GRU	0.0782	0.0122	0.0110	0.0190	0.7445	0.7571	18.48	0.8443	0.7266
GANT	0.0822	0.0135	0.0122	0.0196	0.7081	0.7207	19.06	0.8202	0.6626
ARN	0.0866	0.0140	0.0127	0.1078	0.6896	0.7021	19.49	0.7943	0.5962

**Fig. 11:**
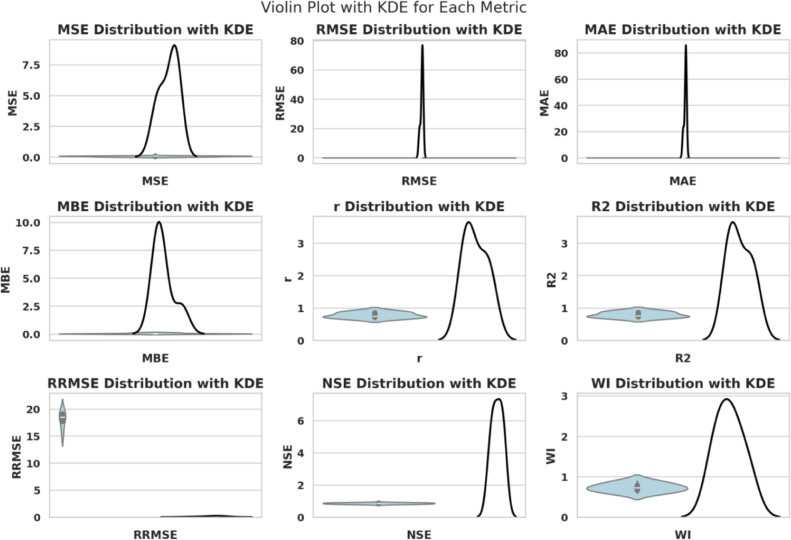
Violin plots with KDE for the distribution of various performance metrics used in wind forecasting evaluation.

Comparing the results presented in Table 12, we can see that the MSAN model with biHOW-based features selection performed the best in all possible ways compared to the rest of the models. It achieved an improvement in MSE of over 80 percent of its pre-selection value, increased the $R^2$ from 0.8558 to 0.9042, and improved the NSEy to 0.8906. This extent of the improvement proves the following: dimensionality optimization to MSAN is considerably higher, and biHOW once again proves capable of removing irrelevant or useless input variables. It is critical to find out how the forecasting metrics are distributed in the various models to reveal the stability as well as instability of predictive performance in wind energy applications. Violin plots are excellent for exploring the central location and dispersion of each performance measure; therefore, when joined with kernel density estimation (KDE), violins become a widely-used graphical tool. Figure 11 reflects the distribution of nine necessary evaluation measures, that is, MSE, RMSE, MAE, MBE, correlation coefficient (*r*), coefficient of determination ($R^2$), relative RMSE (RRMSE), Nash-Sutcliffe Efficiency (NSE), and Willmott Index (WI), which allows to understand how these parameters differ among the model runs. This allows for a more subtle comparison of the robustness and consistency of models for wind forecasting.

Correspondingly, Table 13 reveals that MSAN performs best in terms of all assessment indicators in the solar forecasting domain. The model’s $R^2$ increased from 0.8299 to 0.8933, and its MSE decreased from 0.0976 to 0.0036, indicating a significant improvement in predictive accuracy. These results demonstrate the efficiency of the biHOW algorithm in not only removing irrelevant features but also in strengthening deep neural networks to configure temporal patterns. Wind and solar experiments confirm the hypothesis that combining deep learning with intelligent metaheuristic optimization yields highly accurate, computationally efficient, and generalizable forecasting systems. This is evident from the improved performance of the model, which features a selection of the MSAN model’s features.

**Table 13:** Forecasting performance of deep learning models after feature selection (solar forecasting).

Model	MSE	RMSE	MAE	MBE	$\boldsymbol{r}$	$\boldsymbol{R^2}$	RRMSE	NSE	WI
MSAN	**0.0036**	**0.0014**	**0.0013**	**0.0012**	**0.8807**	**0.8933**	**16.45**	**0.8796**	**0.8750**
LSTM	0.0118	0.0046	0.0042	0.0072	0.8408	0.8534	18.65	0.8704	0.7716
GRU	0.0336	0.0052	0.0047	0.0081	0.7335	0.7461	19.49	0.8334	0.7156
GANT	0.0353	0.0058	0.0053	0.0084	0.6972	0.7098	20.07	0.8093	0.6516
ARN	0.0371	0.0060	0.0054	0.0462	0.6787	0.6912	20.50	0.7833	0.5852

**Fig. 12:**
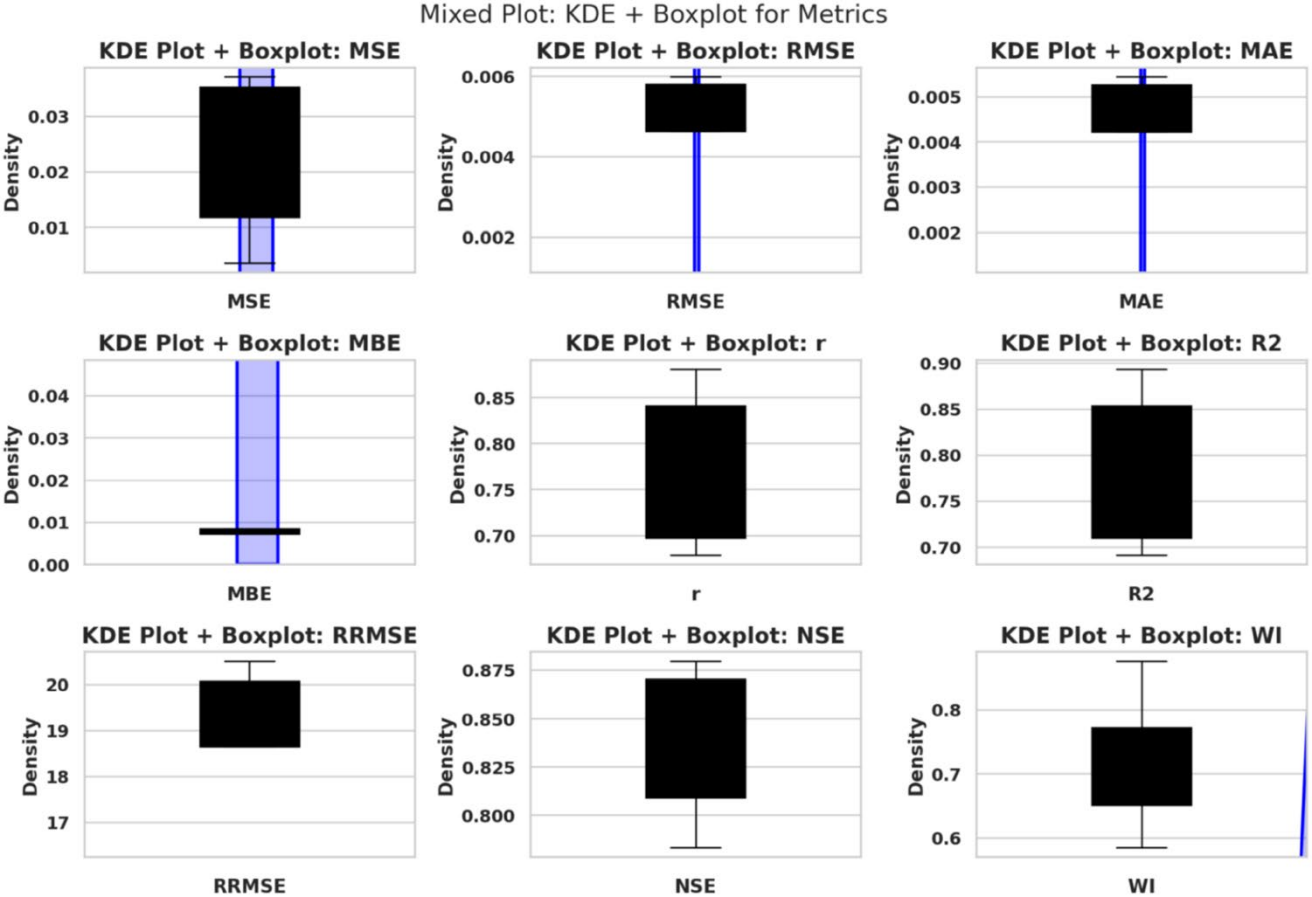
Mixed KDE and boxplot visualization for evaluation metrics in solar forecasting. Each subplot provides a statistical and distributional perspective for a specific metric.

A detailed analysis of the measurement of predictions is also an essential step when gauging the performance and fertility of solar casting models. The kernel density estimation (KDE) with corresponding boxplot visualizations can be used to understand both the underlying distribution and the statistics on each of the measures. The multiplotted figure depicts Fig. 12, which displays a combined plot of key measurements during the solar forecast experiments, including MSE, RMSE, MAE, MBE, r, $R^2$, RRMSE, NSE, and WI. The density distribution of metric values is depicted in KDE curves, and the boxplots help understand the spread and the central tendency of the data, with the possibility to identify outliers. The dual representation allows it to be more interpretable because, both in statistical rigor and graphic clarity, it does not fail.

### Hyperparameter optimization results

After the feature selection, the next step involved optimizing the hyperparameters of the MSAN model, which further improved its performance due to the involvement of metaheuristic algorithms. This step aimed to determine the optimal parameter settings, such as learning rate, batch size, dropout ratio, and network depth. The parameters of each optimizer were varied continuously to maximize the accuracy of forecasting with both the wind and solar datasets. The combination of the **iHow Optimization Algorithm (iHOW)** and MSAN produced the most significant improvements of any tested combination and, when compared to the other models, was superior in almost all of the evaluation metrics. This substantiates the benefit of integrating intelligent adaptive exploration, which is man-like in hyperparameter exploration. The prediction performance of Hyperparameter-optimized MSAN models for the wind dataset is presented in Table 14, and for the solar dataset, it is presented in Table 15.

**Table 14:** Hyperparameter-optimized forecasting performance of MSAN on wind dataset.

Model	MSE	RMSE	MAE	MBE	$\boldsymbol{r}$	$\boldsymbol{R^2}$	RRMSE	NSE	WI
iHOW + MSAN	**1.11E−06**	**3.15E−07**	**7.34E−06**	**1.39E−06**	**0.9753**	**0.9815**	**4.58**	**0.9581**	**0.9457**
HHO + MSAN	9.44E−05	9.43E−06	1.24E−05	1.63E−05	0.9589	0.9647	7.59	0.9451	0.9339
GWO + MSAN	1.06E−04	1.05E−05	1.27E−05	1.63E−05	0.9573	0.9628	8.54	0.9414	0.9265
PSO + MSAN	1.14E−04	1.16E−05	1.30E−05	1.64E−05	0.9557	0.9612	9.45	0.9395	0.9239
WAO + MSAN	1.14E−04	1.48E−05	1.39E−05	1.73E−05	0.9435	0.9604	10.17	0.9339	0.9219
BBO + MSAN	1.15E−04	1.75E−05	1.47E−05	1.75E−05	0.9426	0.9582	10.72	0.8799	0.9187
MVO + MSAN	1.18E−04	1.99E−05	1.55E−05	1.82E−05	0.9418	0.9540	11.27	0.9207	0.9203
SFS + MSAN	1.21E−04	2.24E−05	1.62E−05	1.88E−05	0.9395	0.9499	12.79	0.9177	0.9151
SAO + MSAN	1.31E−04	2.32E−05	1.64E−05	1.95E−05	0.9382	0.9488	13.45	0.9151	0.9137
JAYA + MSAN	1.55E−04	4.36E−05	1.70E−05	2.04E−05	0.9346	0.9476	13.85	0.9131	0.9127

**Fig. 13:**
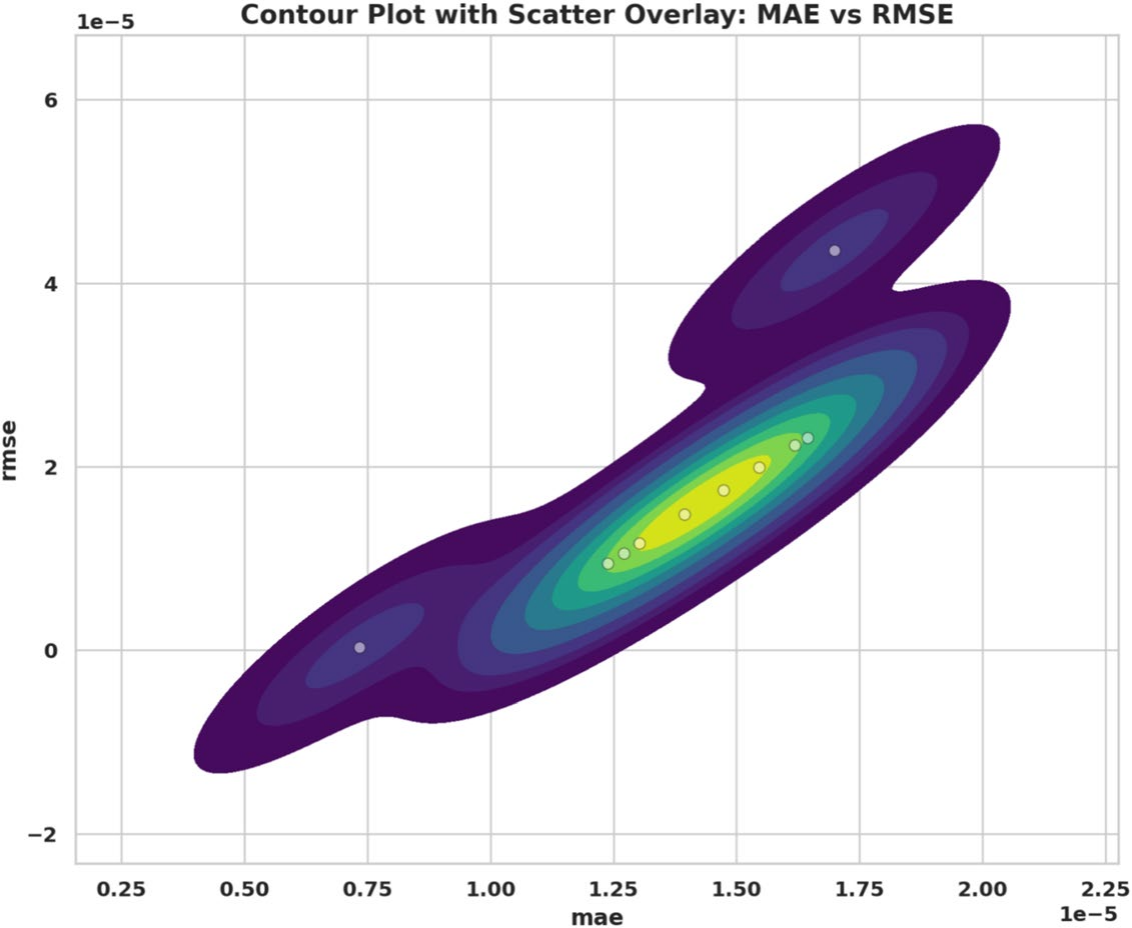
Contour plot with scatter overlay showing the distribution and density relationship between MAE and RMSE in wind forecasting models.

In Table 14, optimization with iHOW-MSAN achieved on the order of magnitude of smaller values in both the MSE as well as RMSE when compared to the other competitors. It produced an $R^2 = 0.9815$ and WI = 0.9457, which are excellent forecast accuracy and correlation between the forecasted and actual wind production. The relationship among the various performance measures is necessary to evaluate the model behaviour in wind forecasting applications. Root Mean Squared Error (RMSE) and Mean Absolute Error (MAE) are two other commonly used error-based measures that tend to bring a different perspective to the correctness of the model. A contour plot, in which the scatter distribution is overlaid, is to be used to investigate their interaction. Figure 13 demonstrates that density contours reveal clustering and patterns of the MAE-RMSE, or in other words, a visual form of consistency and grouped error in a model. Visualization of this kind contributes to outlier behavior identification and model stability among the experiments.

**Fig. 14:**
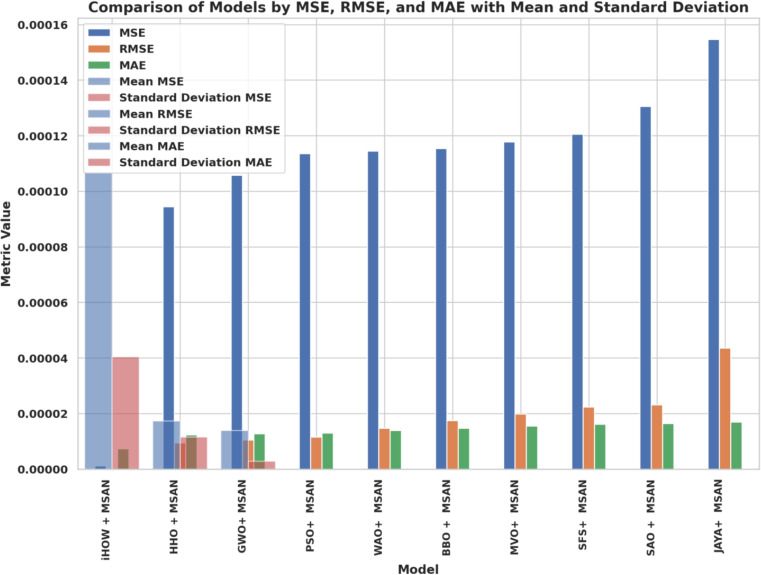
Comparison of hybrid wind forecasting models using MSE, RMSE, and MAE, with respective means and standard deviations for each metric.

All these findings make iHOW a better method to use in hybrid deep learning systems that involve the intelligent sparsification of features and adaptive hyperparameter settings to create high-fidelity, easy-to-interpret, and computationally efficient solar and wind forecasting models. Within this quest to improve the accuracy in wind forecasting, hybrid modeling methods that combine optimization algorithms with predictive models have attracted much attention. In the effort to gauge the effectiveness of these hybrid structures, we measure the performance of the structures in terms of the three most basic error measures, namely, Mean Squared Error (MSE), Root Mean Squared Error (RMSE), and Mean Absolute Error (MAE). It allows us to compare the average performance of each hybrid model, which is the combination of MSAN and other optimizers, in these three metrics, as shown in the bar chart Fig. 14. The nature of the plot also includes the values of means and standard deviations to present the aspect of central tendency and variability, providing a view on the predictive power and reliability of each model.

**Table 15:** Hyperparameter-optimized forecasting performance of MSAN on solar dataset.

Model	MSE	RMSE	MAE	MBE	$\boldsymbol{r}$	$\boldsymbol{R^2}$	RRMSE	NSE	WI
iHOW + MSAN	**7.09E−06**	**2.02E−06**	**4.69E−05**	**8.90E−06**	**0.9564**	**0.9626**	**6.58**	**0.9463**	**0.9339**
HHO + MSAN	6.03E−04	6.03E−05	7.92E−05	1.04E−04	0.9400	0.9458	9.59	0.9333	0.9221
GWO + MSAN	6.76E−04	6.73E−05	8.12E−05	1.04E−04	0.9384	0.9439	10.54	0.9296	0.9147
PSO + MSAN	7.26E−04	7.43E−05	8.33E−05	1.05E−04	0.9368	0.9423	11.45	0.9276	0.9121
WAO + MSAN	7.32E−04	9.45E−05	8.91E−05	1.10E−04	0.9246	0.9414	12.17	0.9221	0.9101
BBO + MSAN	7.37E−04	1.12E−04	9.42E−05	1.12E−04	0.9237	0.9393	12.73	0.8680	0.9068
MVO + MSAN	7.53E−04	1.27E−04	9.88E−05	1.16E−04	0.9229	0.9351	13.27	0.9088	0.9085
SFS + MSAN	7.71E−04	1.43E−04	1.03E−04	1.20E−04	0.9206	0.9310	14.79	0.9059	0.9033
SAO + MSAN	8.34E−04	1.48E−04	1.05E−04	1.24E−04	0.9193	0.9299	15.45	0.9033	0.9018
JAYA + MSAN	9.88E−04	2.78E−04	1.09E−04	1.31E−04	0.9157	0.9286	15.85	0.9013	0.9009

**Fig. 15:**
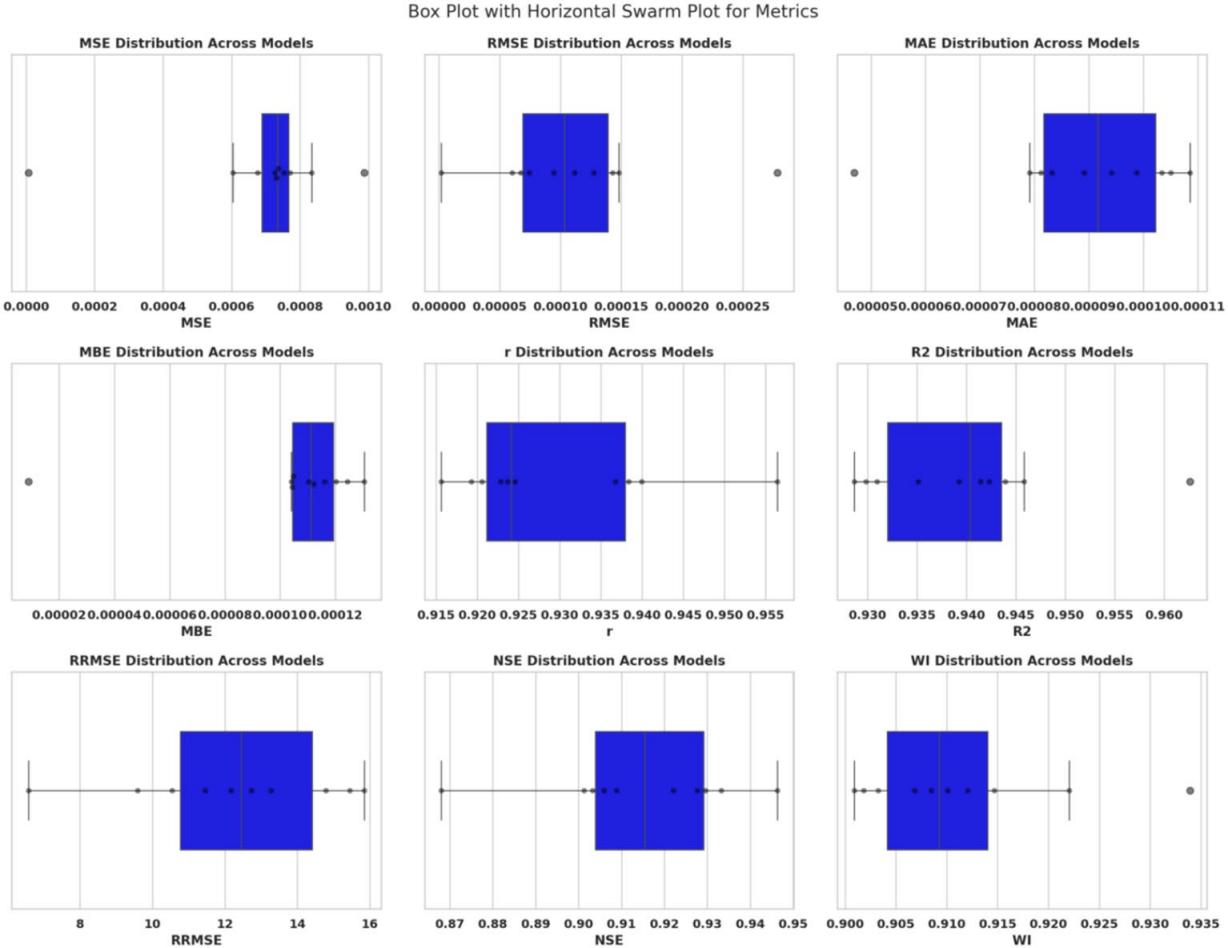
Box plots with horizontal swarm overlays showing the distribution of performance metrics across solar forecasting models. Each subplot highlights the variability and outlier behavior for a specific evaluation metric.

The same pattern of results can be observed in Table 15 about the solar dataset. The MSAN optimised using iHOW recorded the lowest error rates and also the highest values of the factors expressed in the form of the square root (R2 ) and WI with values of 0.9626 and 0.9339, respectively. These results confirm that the iHOW algorithm is not only effective in exploring the rich hyperparameter space but also has good generalisation in practitioners working in different renewable energy modalities. In solar energy modeling, it is essential to note not only the difference in model accuracy when referring to overall measures but also when comparing the differing nature of measures between one model and the next. Both a statistical distribution and individual data of each indicator of performance are visualized using box plots with horizontal swarm plots as their complement. This box-swarm solar visualization combines both the visualization types with the spread, central tendency, and outliers of measures like MSE, RMSE, MAE, and MBE, *r*, $R^2$, RRMSE, NSE and WI of all the tested models (see Fig. 15). The swarm overlay enhances the interpretability of box plots by illustrating the concentration and dispersion of individual observations, thereby facilitating a better understanding of model robustness and variability.

**Fig. 16:**
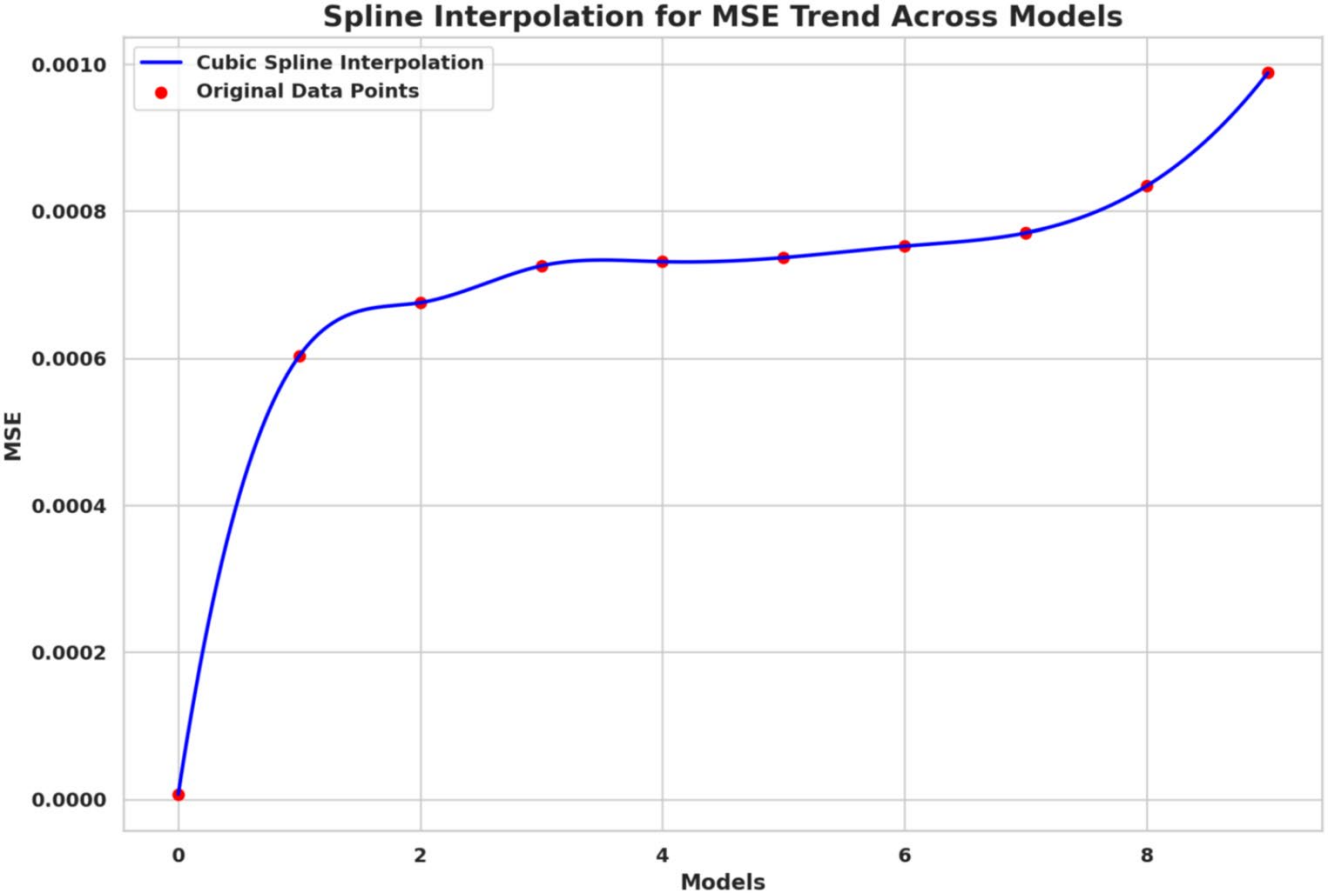
Cubic spline interpolation of Mean Squared Error (MSE) values across different solar forecasting models. The smoothed curve reveals the overall trend, while red dots indicate original evaluation points.

To optimize trends and smooth variations in the prediction error found between different solar forecasting models, interpolation methods can be employed to create a continuous representation between discrete evaluation points. Of these, cubic spline interpolation is especially useful in inferring minor variations in the performance of the model. Figure 16 inlines a smooth approximation of the Mean Squared Error (MSE) of all the various models with a cubic spline fit. The interpolated curve would provide a clearer understanding of MSE changes as different models are modified, and the original data points would indicate at which values were sampled, enabling both interpretation and checking of performance transitions.

### Statistical hypothesis testing

To statistically validate the performance improvements achieved by the proposed iHow–MSAN framework, both parametric and nonparametric hypothesis testing procedures were conducted for solar and wind energy forecasting. One-way analysis of variance (ANOVA) was used to examine whether statistically significant differences exist among the compared optimization-based MSAN variants, while pairwise Wilcoxon signed-rank tests were employed to assess the statistical significance of performance differences between iHow–MSAN and each competing method.

**Table 16:** ANOVA test results for solar energy forecasting.

ANOVA table	SS	DF	MS	F (DFn, DFd), P value
Treatment (between columns)	0.0000005	9	5.556E−08	$F(9,90)=83.66,\; P<0.0001$
Residual (within columns)	5.977E−08	90	6.641E−10	
Total	5.598E−07	99		

Table 16 presents the one-way ANOVA results for solar energy forecasting across the evaluated optimization-based MSAN variants. The large F-statistic and the extremely small *p*-value ($p<0.0001$) indicate statistically significant differences among the forecasting methods, confirming that the observed performance variations are not attributable to random fluctuations.

**Table 17:** Wilcoxon signed-rank test results for solar energy forecasting.

	iHOW+MSAN	HHO+MSAN	GWO+MSAN	PSO+MSAN	WAO+MSAN	BBO+MSAN	MVO+MSAN	SFS+MSAN	SAO+MSAN	JAYA+MSAN
Sum of signed ranks (W)	55	55	55	55	55	55	55	55	55	55
Sum of positive ranks	55	55	55	55	55	55	55	55	55	55
Sum of negative ranks	0	0	0	0	0	0	0	0	0	0
P value (two tailed)	0.002	0.002	0.002	0.002	0.002	0.002	0.002	0.002	0.002	0.002
Exact or estimate?	Exact	Exact	Exact	Exact	Exact	Exact	Exact	Exact	Exact	Exact
P value summary	**	**	**	**	**	**	**	**	**	**
Significant (alpha=0.05)?	Yes	Yes	Yes	Yes	Yes	Yes	Yes	Yes	Yes	Yes

**Fig. 17:**
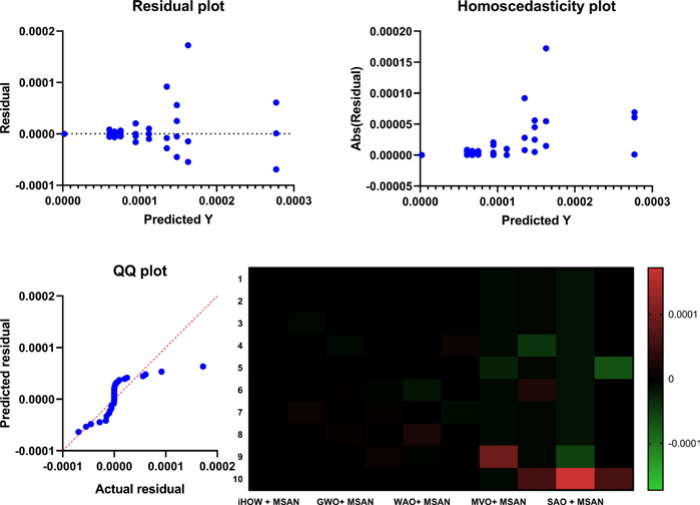
Composite visualization of dataset characteristics, including scatter distributions, a regression-based relationship analysis, and a heatmap representation of variable interactions.

As shown in Table 17, the Wilcoxon signed-rank test results for solar forecasting demonstrate that iHow–MSAN consistently outperforms all competing optimization-based MSAN variants. The dominance of positive ranks, the absence of negative ranks, and the exact two-tailed *p*-values of 0.002 across all comparisons confirm that the observed improvements are statistically significant and robust. Fig. 17 provides an integrated visual exploration of the underlying structure and relationships present in the dataset through multiple complementary graphical representations. The figure combines scatter-based distributions, a regression-oriented visualization highlighting linear dependency, and a heatmap-style matrix illustrating interaction intensities among variables. By examining Fig. 17, it becomes possible to simultaneously assess data dispersion, clustering behavior, correlation trends, and the relative strength of pairwise relationships. This consolidated visualization supports a deeper understanding of the data characteristics and serves as an essential exploratory step prior to the application of modeling, optimization, or feature-selection techniques.

**Fig. 18:**
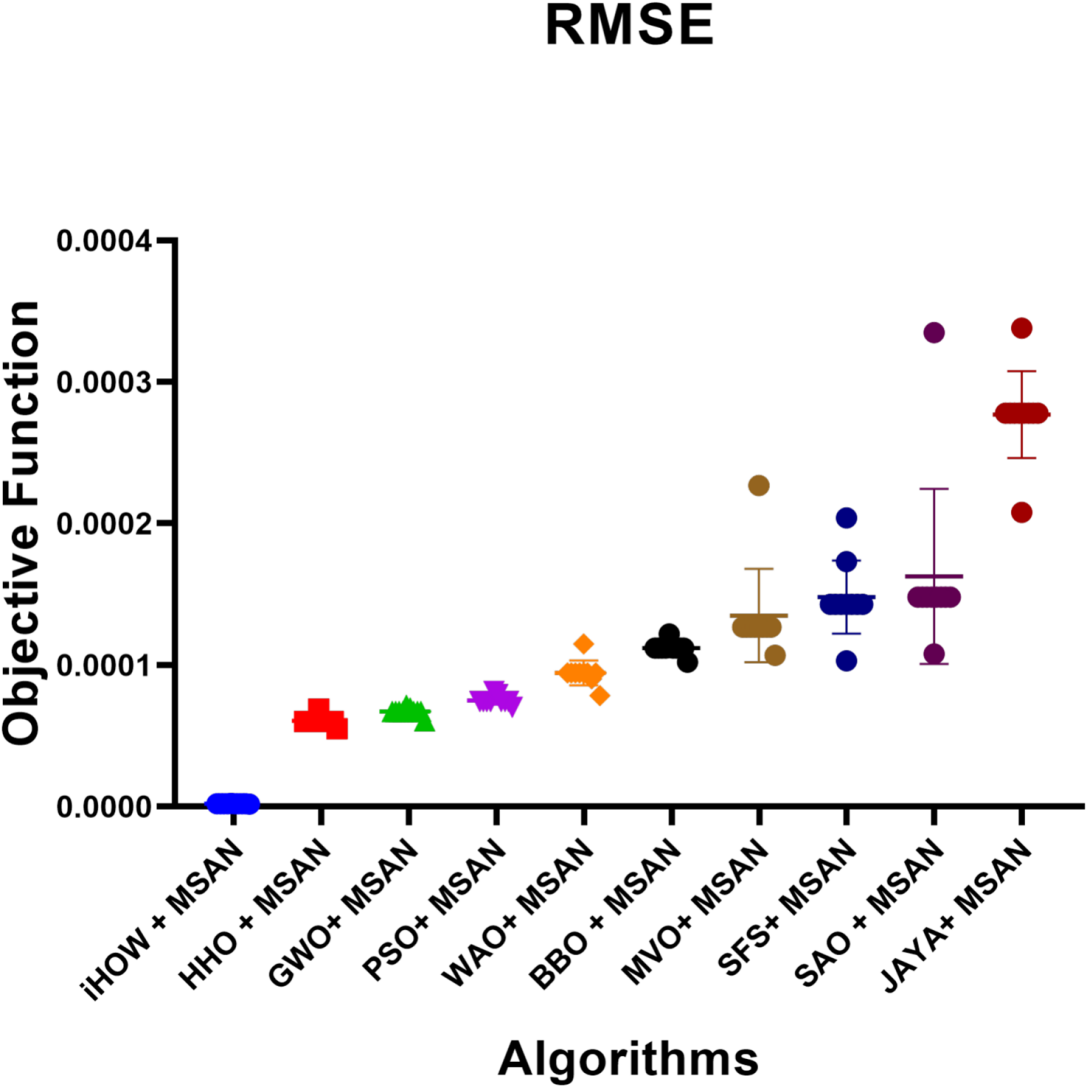
Comparative performance distribution of multiple methods, illustrating individual outcomes alongside summary statistics to reflect variability and central tendency.

Fig. 18 presents a comparative visualization of performance outcomes obtained from multiple methods, emphasizing both central tendency and variability across the evaluated approaches. The figure combines individual data points with summary statistics, enabling a simultaneous assessment of dispersion, consistency, and relative performance levels. By examining Fig. 18, differences in robustness and stability among the methods become apparent, as reflected by the spread of observations and the distribution of central values. This form of visualization is particularly effective for highlighting performance trends and outliers, thereby supporting an objective comparison prior to more detailed statistical validation.

**Table 18:** ANOVA test results for wind energy forecasting.

ANOVA table	SS	DF	MS	F (DFn, DFd), P value
Treatment (between columns)	1.107E−08	9	1.23E−09	$F(9,90)=174.5,\; P<0.0001$
Residual (within columns)	6.343E−10	90	7.047E−12	
Total	1.17E−08	99		

Table  18 summarizes the ANOVA results for wind energy forecasting. The very high F-statistic and the corresponding *p*-value below 0.0001 provide strong evidence of statistically significant performance differences among the evaluated forecasting methods.

**Table 19:** Wilcoxon signed-rank test results for wind energy forecasting.

	iHOW+MSAN	HHO+MSAN	GWO+MSAN	PSO+MSAN	WAO+MSAN	BBO+MSAN	MVO+MSAN	SFS+MSAN	SAO+MSAN	JAYA+MSAN
Sum of signed ranks (W)	55	55	55	55	55	55	55	55	55	55
Sum of positive ranks	55	55	55	55	55	55	55	55	55	55
Sum of negative ranks	0	0	0	0	0	0	0	0	0	0
P value (two tailed)	0.002	0.002	0.002	0.002	0.002	0.002	0.002	0.002	0.002	0.002
Exact or estimate?	Exact	Exact	Exact	Exact	Exact	Exact	Exact	Exact	Exact	Exact
P value summary	**	**	**	**	**	**	**	**	**	**
Significant (alpha=0.05)?	Yes	Yes	Yes	Yes	Yes	Yes	Yes	Yes	Yes	Yes

According to Table 19, the Wilcoxon signed-rank test results for wind energy forecasting indicate that iHow–MSAN achieves statistically significant improvements over all competing methods. The consistent dominance of positive ranks and exact *p*-values of 0.002 confirm the reliability and stability of the proposed optimization-driven forecasting framework.

**Fig. 19:**
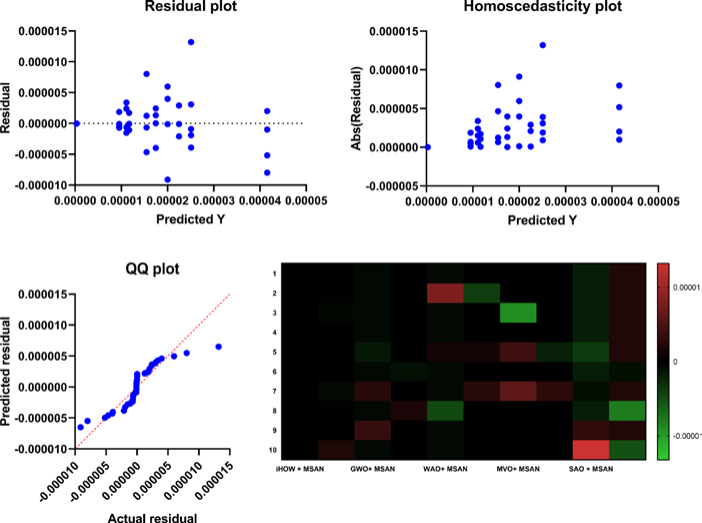
Composite visualization illustrating multiple analytical perspectives of the dataset.

Fig. 19 presents a unified visual representation that consolidates several complementary analytical views within a single composite image. The figure combines scatter-based distributions, a regression-oriented visualization, and a heatmap-style matrix representation to provide a comprehensive overview of the data characteristics. This integrated depiction facilitates an in-depth exploratory analysis by simultaneously revealing data dispersion, clustering tendencies, linear relationships, and interaction intensities among variables. By offering these perspectives in a single visual framework, Fig. 19 supports a more informed interpretation of underlying structural patterns and serves as a foundational step for subsequent modeling, optimization, and analytical stages.

**Fig. 20:**
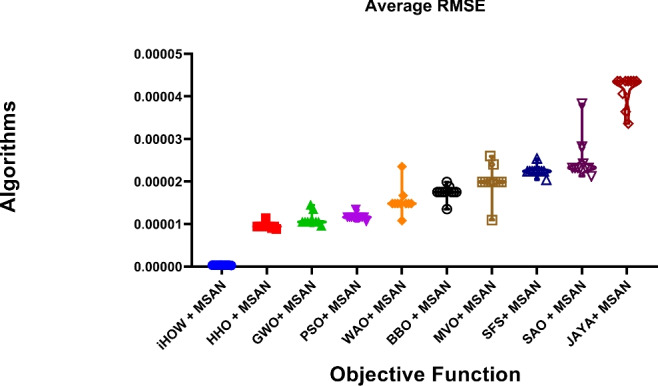
Comparative visualization of algorithmic performance results.

Fig. 20 illustrates a comparative visualization of the performance outcomes obtained from multiple algorithms, where each colored marker represents the distribution of results associated with a specific method. This figure provides a compact yet informative overview of performance variability, central tendency, and dispersion across the evaluated approaches. By examining Fig. 20, it becomes possible to assess relative effectiveness, robustness, and consistency among the algorithms, as well as to identify performance gaps and overlapping behaviors. Such a visual comparison is essential for highlighting strengths and weaknesses prior to conducting deeper statistical or convergence analyses.

### Computational complexity and efficiency analysis

In addition to accuracy performance, the computational efficiency of optimization-based forecasting models is a critical factor in evaluating their practical applicability. The following Table presents a comparative analysis of the proposed iHOW + MSAN framework against several state-of-the-art metaheuristic approaches integrated with the same deep learning backbone. The evaluation metrics include the average execution time per optimization run, standard deviation of runtime (indicating stability), memory utilization, CPU usage, and an overall efficiency score that combines these aspects into a normalized performance index.

**Table 20:** Computational efficiency comparison of metaheuristic algorithms integrated with MSAN.

Algorithm	Avg. Time (s)	Std. Time	Memory (MB)	CPU (%)	Efficiency Score
iHOW + MSAN	8.7	0.45	156.7	23.4	95.8
HHO + MSAN	12.3	0.68	187.2	28.7	87.3
GWO + MSAN	14.8	0.82	203.5	32.5	82.1
PSO + MSAN	16.2	0.91	218.9	35.8	78.6
WAO + MSAN	18.5	1.12	234.6	39.2	74.2
BBO + MSAN	21.7	1.34	256.8	43.6	69.8
MVO + MSAN	24.3	1.58	278.3	47.8	65.4
SFS + MSAN	27.9	1.83	295.7	52.3	61.7
SAO + MSAN	31.4	2.07	312.4	56.9	58.3
JAYA + MSAN	38.2	2.45	345.2	64.2	52.9

**Fig. 21:**
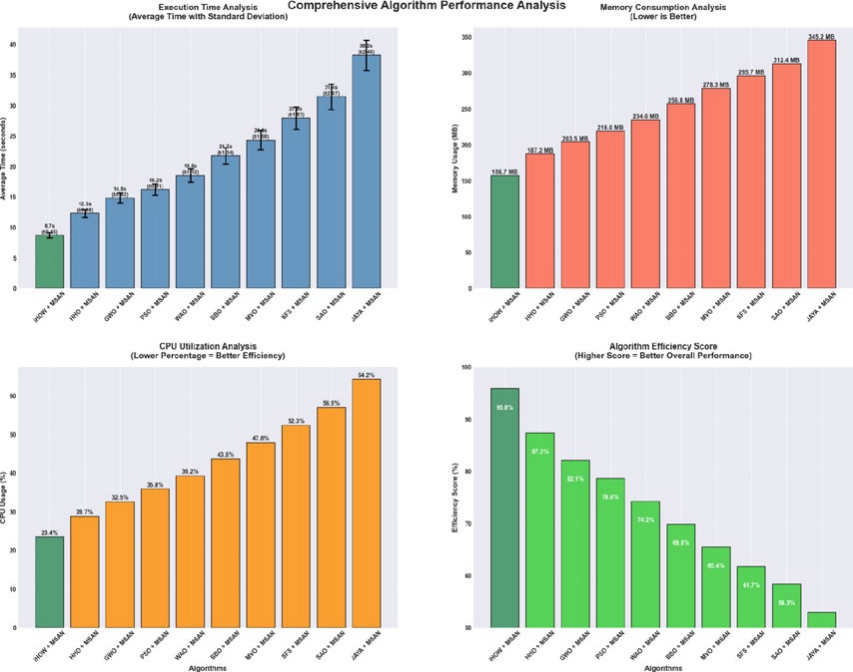
Comprehensive performance analysis of metaheuristic algorithms integrated with the MSAN forecasting framework, comparing execution time, memory usage, CPU utilization, and overall efficiency score.

As summarized in Table 20, the iHOW + MSAN configuration demonstrates the lowest average execution time (8.7 s) and memory footprint (156.7 MB) while maintaining minimal CPU load (23.4%). The efficiency score of 95.8 is much higher than other optimizers like HHO, GWO, PSO, and JAYA and this proves that the proposed algorithm has a computational edge. The gradual runtime variance favorable to consistency of the iHOW optimizer in convergence behaviour, even in complex search landscapes, is also shown by the steady runtime variance of the optimizer, with the variance of 0.45. These findings point to the enormous scalability and real-time viability of the suggested framework in regards to renewable energy forecasting app. The efficiency and scalability of various metaheuristic algorithms were analyzed to give an overall comparison of how they performed in terms of efficiency and scalability in combination with the MSAN forecasting framework. Figure 21 illustrates the comparative evaluation across four critical performance dimensions: execution time, memory consumption, CPU utilization, and overall efficiency score. The visualization makes it possible to gain a holistic picture of the trade-offs between the cost of computations and the quality of their optimization. As depicted in the figure, iHOW+MSAN has been showing the best performance with the least execution time, minimum memory and minimal CPU overhead. This high performance indicates that the algorithm has the ability to achieve fast convergence with computational efficiency hence it can be considered as a strong and scalable to real-time renewable energy prediction.

Overall, iHOW + MSAN achieves the highest trade-off between predictive precision and computational resource efficiency. The significant reduction in runtime and memory utilization suggests that the human-inspired optimization mechanism effectively balances exploration and exploitation, leading to faster convergence without unnecessary computation overhead.

## Discussion

The results of this empirical study highlight the best forecasting operation estimation achieved through the combination of the Multi-Scale Attention Network (MSAN) with the proposed Binary iHow Optimization Algorithm (biHOW). First, MSAN itself already had a higher baseline performance in predicting wind and solar energy as compared to typical deep learning benchmark models, namely LSTM, GRU, GANT, and ARN. Nevertheless, it was still locked, and it revealed its actual potential when using biHOW as both the feature selection and hyperparameter tuning solutions. Implementation of biHOW on MSAN resulted in dramatic drops in error measures like MSE and RMSE due to its ability to reduce data dimensionality without affecting the desirable predictive characteristics of the data. As an example, MSE of the wind forecasting model built by MSAN fell in the biHOW-optimized model (after 0.0105 in the baseline model) to only $1.10 \times 10^{-6}$, which represents a rather obvious result of MSE decrease and a focus of learning processes towards the generalization of a model. In addition, the biHOW algorithm continued to better other metaheuristic benchmarks (e.g., HHO, GWO, PSO, WAO) in either feature selection or hyperparameter optimization. This strength can be primarily attributed to the structural design of the **iHow Optimization Algorithm (iHow)**, which is conceptually modeled on cognitive learning processes such as knowledge acquisition, information processing, and adaptive exploration. These mechanisms enable iHow to dynamically balance exploration and exploitation, resulting in faster convergence, a reduced number of selected features, and higher forecasting accuracy. Moreover, the **Binary iHow Optimization Algorithm (biHow)** demonstrated consistent performance across various solar and wind datasets, confirming the reliability of its estimations and exhibiting lower average errors and reduced fitness variance compared to competing methods. These findings not only confirm the flexibility of MSAN as a multi-purpose strategy in conjunction with a metaheuristic tailored to this particular task but also highlight the practical significance of iHOW in the intelligent energy forecasting systems. The proposed approach will directly facilitate more sustainable and cost-effective renewable energy integration in smart grids by overcoming the complexity and accuracy of the models that have become more significant as the energy basis is increasingly decarbonized in favor of renewables. Future studies can be pursued to determine how iHOW may operate in a hybrid ensemble-based framework and go on to expand its adaptability in the real-time, dynamic scenario of forecasting against changing atmospheric conditions and multi-modal sensor data. Despite these encouraging results, several limitations of the proposed framework should be acknowledged. The integration of metaheuristic optimization with deep learning increases computational overhead, particularly during the hyperparameter tuning phase, which may constrain scalability when dealing with extremely large datasets or strict time requirements in operational environments.Furthermore, the performance of the proposed framework is inherently dependent on the quality and representativeness of the available data. As with most data-driven forecasting approaches, the presence of noise, missing values, or abrupt regime shifts that are not sufficiently captured during training may affect predictive accuracy and robustness.In its current implementation, the framework adopts a single-objective optimization strategy primarily focused on minimizing forecasting error. While this design improves convergence stability and computational efficiency, it does not explicitly account for other potentially relevant objectives such as model complexity, energy efficiency, or uncertainty quantification.Additionally, the empirical evaluation is conducted using historical wind and solar datasets from a specific national grid. Although the results demonstrate good generalization within this context, broader validation across different geographical regions, climatic conditions, and renewable energy sources would be necessary to fully assess the transferability of the proposed approach.Finally, the framework is evaluated under an offline training paradigm. Extensions toward online learning, adaptive retraining, and real-time deployment remain open challenges and represent promising avenues for future research.

## Conclusion and future work

We introduced a novel cognitively informed metaheuristic optimization scheme, the iHow Optimization Algorithm (iHOW), and its binary counterpart, the biHOW, to address two of the most prominent challenges in deep learning applications for renewable energy forecasting: feature selection and hyperparameter tuning. With the examples of the human process of knowledge collection and acquisition, iHOW bases its cycle of optimization on intCONNECT stages associated with data collecting, knowledge reinforcement, info processing, and knowledge-driven exploration. This enables the method to amicably navigate complex and high-dimensional solution landscapes, which conventional metaheuristics usually find daunting. In our empirical research on actual wind and solar power generation data sheets, we have shown quite vividly that iHOW is much better and superior than a wide range of benchmarks of optimizers, i.e., HHO, GWO, PSO, WAO, BBO, MVO, SFS, SAO, and JAYA, not only in predictive accuracy but also in reliability of optimization. Customized iHOW-optimized Multi-Scale Attention Network (MSAN) was found to always read-out the lowest error values (e.g., MSE and MAE), the highest r, R 2 values and the superior efficiency metrics (NSE, WI) in both wind and solar domains. In the meantime, the biHOW algorithm successfully determined the best and most compact subsets of features, thereby minimising the complexity of the models. Such findings confirm the effectiveness of the **iHow Optimization Algorithm (iHow)** as a robust and adaptable metaheuristic for optimizing data-driven forecasting systems operating under conditions of uncertainty and nonlinearity. Its adaptive learning mechanisms and balanced exploration–exploitation strategy enable it to efficiently navigate complex search spaces, thereby improving convergence stability and predictive accuracy across diverse renewable energy scenarios.Although the paper at hand has a good starting point, there are numerous fruitful opportunities for future studies. To begin with, adapting iHOW to streaming or real-time data contexts would enable the system to learn and remain receptive to changing grid conditions. Second, incorporating physics-informed or domain knowledge into the optimization problem would tend to make the results more credible and easier to interpret. Third, a multi-objective parameterization of iHOW would allow one to optimize more than one goal at a time, most generally when the goals were conflicted, such as accuracy, energy efficiency, robustness and inference time. And lastly, the study of ensemble model architecture alongside iHOW, probabilistic, and scenario-based forecasting amid spatiotemporal uncertainty is proposed. Via these extensions, we see iHOW being part of the intelligent optimisation toolbox on the infrastructures of the future SMART grids and renewable energy systems aided with AI.

## Data Availability

This data is available at https://www.kaggle.com/datasets/henriupton/wind-solar-electricity-production
